# Heterogeneity of response to immune checkpoint blockade in hypermutated experimental gliomas

**DOI:** 10.1038/s41467-020-14642-0

**Published:** 2020-02-18

**Authors:** Katrin Aslan, Verena Turco, Jens Blobner, Jana K. Sonner, Anna Rita Liuzzi, Nicolás Gonzalo Núñez, Donatella De Feo, Philipp Kickingereder, Manuel Fischer, Ed Green, Ahmed Sadik, Mirco Friedrich, Khwab Sanghvi, Michael Kilian, Frederik Cichon, Lara Wolf, Kristine Jähne, Anna von Landenberg, Lukas Bunse, Felix Sahm, Daniel Schrimpf, Jochen Meyer, Allen Alexander, Gianluca Brugnara, Ralph Röth, Kira Pfleiderer, Beate Niesler, Andreas von Deimling, Christiane Opitz, Michael O. Breckwoldt, Sabine Heiland, Martin Bendszus, Wolfgang Wick, Burkhard Becher, Michael Platten

**Affiliations:** 1 DKTK Clinical Cooperation Unit Neuroimmunology and Brain Tumor Immunology, German Cancer Research Center (DKFZ), Heidelberg, Germany; 2 Department of Neurology, Medical Faculty Mannheim, MCTN, Heidelberg University, Heidelberg, Germany; 3 Faculty of Biosciences, Heidelberg University, Heidelberg, Germany; 4 Institute of Experimental Immunology, University of Zurich, Zurich, Switzerland; 5 Department of Neuroradiology, Heidelberg University Medical Center, Heidelberg, Germany; 6 Brain Cancer Metabolism Group, German Cancer Research Center (DKFZ), Heidelberg, Germany; 7 DKTK Clinical Cooperation Unit Neuropathology, German Cancer Research Center (DKFZ), Heidelberg, Germany; 8 Department of Neuropathology, Heidelberg University Medical Center, Heidelberg, Germany; 9 nCounter Core Facility, Institute of Human Genetics, University of Heidelberg, Heidelberg, Germany; 10 Department of Neurology, Heidelberg University Medical Center, Heidelberg, Germany; 11 National Center for Tumor Diseases Heidelberg, DKTK, Heidelberg, Germany; 12 DKTK CCU Neurooncology, DKFZ, Heidelberg, Germany; 13 Helmholtz Institute for Tranlational Oncology (HI-TRON), Mainz, Germany; 14 Present Address: Immatics Biotechnologies GmbH, Tübingen, Germany; 15 Present Address: Institute of Neuroimmunology and Multiple Sclerosis, Center for Molecular Neurobiology Hamburg, University Medical Center Hamburg-Eppendorf, Hamburg, Germany

**Keywords:** Cancer models, Neuroimmunology, Immunosurveillance

## Abstract

Intrinsic malignant brain tumors, such as glioblastomas are frequently resistant to immune checkpoint blockade (ICB) with few hypermutated glioblastomas showing response. Modeling patient-individual resistance is challenging due to the lack of predictive biomarkers and limited accessibility of tissue for serial biopsies. Here, we investigate resistance mechanisms to anti-PD-1 and anti-CTLA-4 therapy in syngeneic hypermutated experimental gliomas and show a clear dichotomy and acquired immune heterogeneity in ICB-responder and non-responder tumors. We made use of this dichotomy to establish a radiomic signature predicting tumor regression after pseudoprogression induced by ICB therapy based on serial magnetic resonance imaging. We provide evidence that macrophage-driven ICB resistance is established by CD4 T cell suppression and T_reg_ expansion in the tumor microenvironment via the PD-L1/PD-1/CD80 axis. These findings uncover an unexpected heterogeneity of response to ICB in strictly syngeneic tumors and provide a rationale for targeting PD-L1-expressing tumor-associated macrophages to overcome resistance to ICB.

## Abstract (web summary)

Modeling patient-individual resistance to immunotherapy is challenging. Here, the authors use a syngeneic experimental hypermutated orthotopic glioma model to define radiological and biological features that can predict or explain the mechanistic differences between responders and non-responders to immunotherapy.

## Introduction

Blockade of immune-regulatory receptors, such as programmed cell death protein-1 (PD-1) and cytotoxic T-lymphocyte-associated antigen-4 (CTLA-4) mitigates T cell suppression, and restores T cell activation and proliferation, thereby reinvigorating antitumor immunity^1^. Immune checkpoint blockade (ICB) targeting PD-1 and CTLA-4 is now implemented into the standard therapies of an increasing number of tumor entities, resulting in durable responses and increased survival in a substantial number of patients^2,3^. While efficacy in metastatic disease to the brain indicates that the central nervous system (CNS) is not a general barrier for ICB-mediated stimulation of antitumor immunity^4,5^, evidence from randomized clinical trials suggest that primary malignant brain tumors, such as glioblastoma are largely resistant with few hypermutated glioblastoma, representing an exception^6,7^. Hypermutation in glioblastomas is not strictly associated with an increased intratumoral T cell response^8–10^, indicating that hypermutation per se is not sufficient for an effective antitumor immunity induced by ICB. Contrariwise, durable responses may occur in patients with glioblastoma (GBM) without hypermutation^11^. Due to the overall low response rate with very few patients responding, both the establishment of predictive biomarkers and the identification of resistance mechanisms is challenging. Syngeneic orthotopic glioblastoma models have been considered insufficient models to assess interindividual heterogeneity of immune responses. To evaluate mechanisms of response and resistance to ICB, we made use of a syngeneic experimental hypermutated orthotopic glioma model exceeding 100 non-synonymous mutations per tumor exome^12–14^ to ensure sufficient immune recognition of neo-epitopes.

Here, we made use of the dichotomy of response and non-response to ICB in a hypermutated glioma model to develop a predictive radiomic imaging signature and to uncover cellular and molecular mechanisms of response and non-response in the glioma immune microenvironment, providing a rationale for targeting programmed death-ligand 1 (PD-L1)-expressing tumor-associated macrophages to overcome resistance to ICB.

## Results

### Preclinical MRI-based response evaluation for GBM immunotherapy

**Fig. 1:**
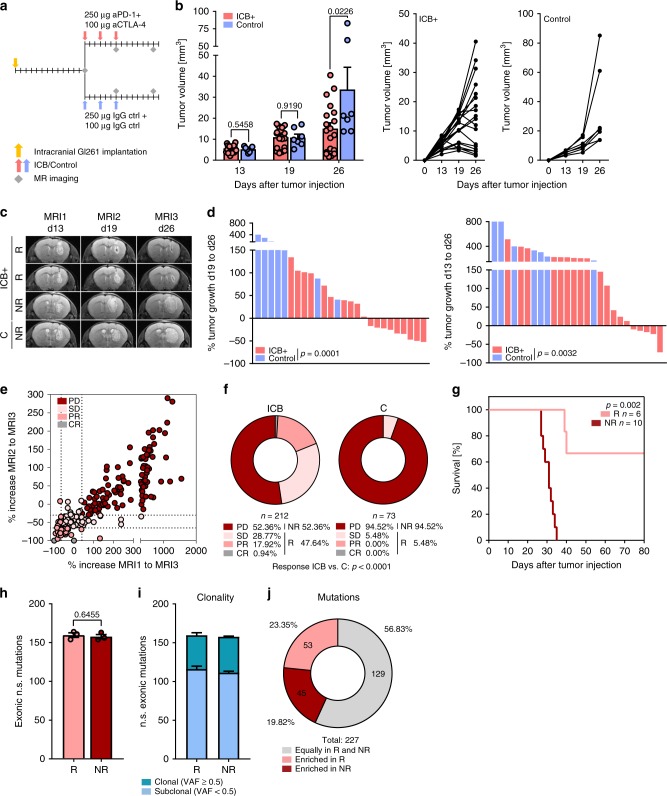
PD-1 and CTLA-4 blockade decreases Gl261 tumor growth in ICB R mice. **a**–**d** C57Bl/6 J mice were treated with 250 µg anti-PD-1 and 100 µg anti-CTLA-4 (ICB+) or isotype control (C) on d13, d16, and d19. Tumor growth was monitored by MRI on d13 (MRI1), d19 (MRI2), and d26 (MRI3) post intracranial Gl261 injection (*n* = 19 vs. *n* = 7 animals). **b**, **c** Tumor growth and representative MR images of ICB+responder (R), non-responder (NR), and control-treated (C) mice. **d** Response assessed by % of tumor growth between d19 and d26, and between d13 and d26 post tumor inoculation. **e**, **f** Advanced response evaluation was performed on an extended dataset (ICB *n* = 212 vs. C *n* = 73 animals). Mice were grouped according to their response pattern with complete response (CR): %V_MRI3–MRI1_ −100 %, partial response (PR): % V_MRI3-MRI1_ ≤ −65.0 % or % V_MRI3-MRI2_ ≤ −65.0 %, sfig disease (SD): %*V*_MRI3–MRI1_ > −65.0% and < + 40.0% or %*V*_MRI3–MRI1_ ≥ + 40.0% and %*V*_MRI3–MRI2 _≤ −30% and progressive disease (PD): %*V*_MRI3–MRI1_ ≥ + 40.0%. **e** Relative increase in lesion volume MRI1–MRI3 (%*V*_MRI3–MRI1_) vs. relative increase in lesion volume MRI2–MRI3 (%*V*_MRI3–MRI2_). **f** Response pattern of ICB and C mice. **g** Survival of ICB R and ICB NR mice (*n* = 6 vs. *n* = 10 animals). Data of two independent experiments were pooled. **h** Tumors of ICB R and ICB NR mice were excised on d26 post tumor inoculation and exonic non-synonymous (n.s.) mutational load was assessed by exome sequencing (*n* = 3 vs. *n* = 3 animals). **i**, **j** Clonality of mutations in ICB R and ICB NR tumors **i** and mutations predominantly enriched in ICB R or ICB NR tumors, VAF, variant allele frequency. **j** Data are represented as mean ± SEM for **b**, **h** and **i**. Statistical significance was determined by two-tailed Student’s *t*-test for **b**, **d** and **h**, Fisher’s exact test for **f** or log-rank Mantel–Cox test for **g**. Source data are provided as a Source Data file.

Combination ICB therapy targeting PD-1 and CTLA-4 suppressed tumor growth of established syngeneic orthotopic mouse gliomas (Fig. 1a–d). Despite strict syngeneity of the model, we observed a dichotomy in tumor growth upon ICB therapy in ICB responder (R) and non-responder (NR) mice as monitored by serial magnetic resonance imaging (MRI; Fig. 1b, d). To evaluate the dynamics of response and resistance in individual mice, we defined preclinical MRI response criteria based on the established clinical RANO (response assessment in neuro-oncology) critera^15^. ICB response in the preclinical model was determined by the comparison of d13 baseline lesion volumes (MRI1) with d26 post therapy lesion volumes (MRI3) using T2-weighted MRI. Assessment of lesion volumes (*V*) and their relative increase between d13 and d26, as well as d19 (MRI2, during ICB therapy) and d26 strongly correlated with the assessment of the lesion bidimensional diameter product (area) used in RANO criteria (Supplementary Fig. 1a, b). We next aimed at translating planumetric RANO criteria to tridimensional (volumetric) response criteria by correcting for area–volume divergence (Supplementary Fig. 1c). For tridimensional response criteria, complete response (CR) was defined as relative change in lesion volume MRI3–MRI1 (%*V*_MRI3–MRI1_) of −100%, partial response (PR) as %*V*_MRI3–MRI1_ ≤ −65.0% and/or %*V*_MRI3–MRI2_ ≤ −65.0%, stable disease (SD) as %*V*_MRI3–MRI1_ > −65.5% and < + 40%, and progressive disease (PD) as %*V*_MRI3–MRI1_ ≥ + 40%. Lesions with an unconfirmed progression, defined by a %*V*_MRI3–MRI1_ ≥ + 40% that showed a regression of at least −30% between MRI2 and MRI3 (%*V*_MRI3–MRI2_) were classified as SD (Fig. 1e, Supplementary Fig. 1c, right). Taking the rapid tumor progression of Gl261 tumors into account, mice with CR, PR, and SD were grouped as ICB R and mice with PD were defined as ICB NR. Response evaluation of a dataset of 212 ICB-treated (ICB) and 73 control-treated (C) mice revealed a response rate of 47.64% (ICB) compared to 5.48 % (C; Fig. 1f, *p* > 0.001). Monotherapy with PD-1 blockade showed a reduced response rate (33.33%, Supplementary Fig. 1d–g) compared to anti-PD-1 and anti-CTLA-1 combination therapy as previously described^16^. ICB response evaluation based on MRI data translated into a significantly enhanced survival in ICB R mice (Fig. 1g). Mutanome analysis of ICB R and ICB NR tumors revealed no significant difference in the number and clonality of mutations with a sufficient number of putative neo-antigens to induce tumor immunity (Fig. 1h–j). The mutanome of ICB R and ICB NR tumors was heterogeneous with 23.35% of all identified mutations enriched in ICB R tumors and 19.82% of all identified mutations enriched in ICB NR tumors (Fig. 1j). Although initial tumor size weakly correlated with therapy response (Supplementary Fig. 2a), response was not restricted to small pretreatment tumor volumes and was independent of preexisting, environmental, and genetic factors, including housing or gender (Supplementary Fig. 2b, c). Notably, heterogeneity of response to ICB therapy was not restricted to experimental gliomas but also occurred in experimental syngeneic B16 melanomas (Supplementary Fig. 3a).

### Radiomic evaluation of ICB response and pseudoprogression

**Fig. 2:**
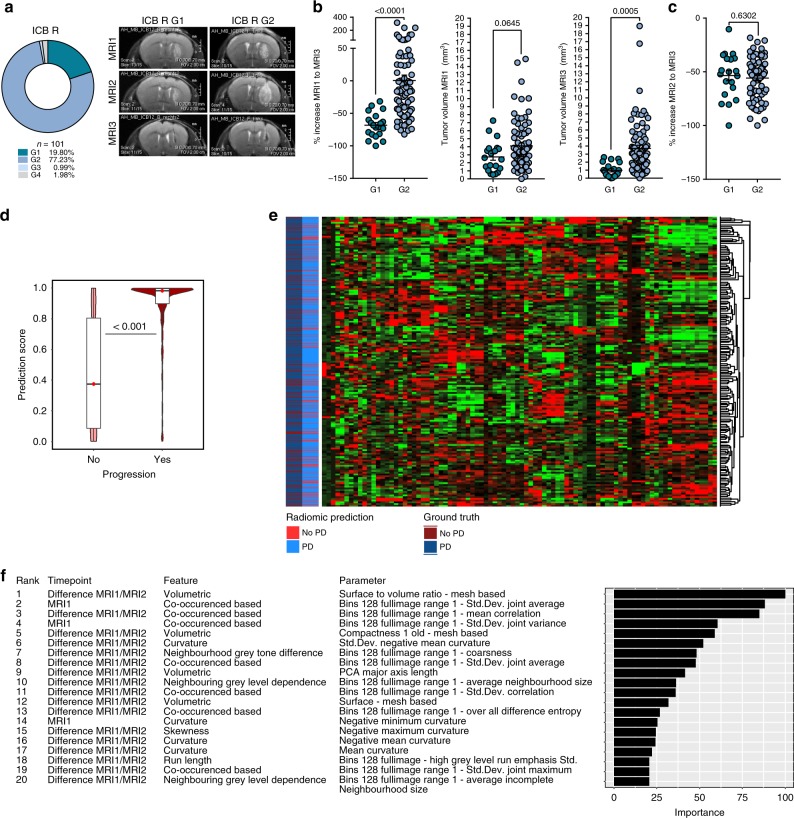
Radiomic prediction of therapy response to ICB therapy. **a**–**c** C57Bl/6 J mice were treated with 250 µg anti-PD-1 and 100 µg anti-CTLA-4 (ICB+) and tumor growth, therapy response and pseudoprogression were evaluated by MRI before (MRI1), during (MRI2), and after ICB therapy (MRI3). **a** Growth pattern analysis of ICB R (*n* = 101 animals). G1: %*V*_MRI2–MRI1_ < 0% and %*V*_MRI3–MRI2_ < 0%; G2: %*V*_MRI2–MRI1_ > 0% and %*V*_MRI3–MRI2_ < 0%; G3: %*V*_MRI2–MRI1_ > 0% and %*V*_MRI3–MRI2_ > 0%; and G4: %*V*_MRI2–MRI1_ < 0% and %*V*_MRI3–MRI2_ > 0%. **b** %*V*_MRI3–MRI1_ (left), *V*_MRI1_ (baseline tumor volume; middle), and *V*_MRI3_ (final tumor volume; right) of ICB R mice with growth pattern G1 and G2 (G1 *n* = 20 vs. G2 *n* = 78 animals). **c** %*V*_MRI3–MRI2_ of ICB R mice with growth pattern G1 and G2 (G1 *n* = 20 vs. G2 *n* = 78 animals). **d**–**f** Radiomic response prediction after ICB therapy based on radiomic features of MRI1 (baseline) and MRI2 (during ICB therapy) images (*n* = 148 animals). Boxplot with blocks showing the interquartile range (IQR) of data points and horizontal central line (red dot) corresponding to the median. The superimposed violin plot visualizes the distribution of the data and its probability density. Radiomic signature score **d**, heatmap of radiomic features **e**, and top predictive radiomic features **f** of R and NR tumors based on radiomic featur**e**s of MRI1 and MRI2. Data are presented as mean ± SEM for **b** and **c**. Statistical significance was determined by two-tailed Student’s *t*-test for **b**–**d**. Source data are provided as a Source Data file.

In serial MRI, we observed evidence of pseudoprogression, where ICB therapy induced an initial increase of the measurable MR lesion between MRI1 and MRI2 followed by a rapid regression between MRI2 and MRI3 in 77.23% of ICB R mice (growth pattern 2; G2), while only 19.80% of ICB R mice showed immediate lesion regression between MRI1 and MRI2 or response between MRI2 and MRI3 without pseudoprogression (G1; Fig. 1c; Fig. 2a). Delayed response of ICB-treated mice with pseudoprogression (G2 growth pattern) resulted in significant bigger tumor volumes on MRI3 compared to directly responding mice (G1 growth pattern; Fig. 2b). However, no significant difference in response between MRI2 and MRT3 was present between G1 and G2 ICB R (Fig. 2c), suggesting that direct response is not a prerequisite for optimal ICB response and methods to monitor pseudoprogression in ICB R are relevant to distinguish pseudoprogressing ICB R from ICB NR.

To non-invasively predict treatment response (R vs. NR) and pseudoprogression in ICB-treated mice, we implemented an MRI-based radiomic approach. We calculated a set of 423 radiomic features from the T2-hyperintense tumor volume for each time point and incorporated features from MRI1 and the change in radiomic features between the MRI1 to MRI2 for radiomic signature discovery. By constructing a gradient boost classifier, we identified a radiomic signature that allowed to predict treatment response with an accuracy of 82.7% (95% confidence interval, 79.8–85.4%; sensitivity: 69.8%; and specificity: 89.9%). Predictive accuracy was significantly higher as compared to the null model (no information rate of 64.2%) (*P* < 0.001; Fig. 2d, e, Supplementary Fig. 3b). The top radiomic feature for prediction of therapy failure to ICB therapy was the shift in the volume to surface ratio between MRI1 and MRI2 (Fig. 2f). As diagnosis, tumor imaging and response monitoring for glioblastoma patient is routinely performed with T1−weighted contrast-enhanced MRI, response evaluation by T2-weighted imaging was validated by simultaneous T1−weighted contrast-enhanced (CE) imaging. T2 and T1 CE tumor volumes strongly correlated (*R*² 0.96; Supplementary Fig. 3c), and T1 CE measurements did not provide an additional benefit for response prediction in pseudoprogressing ICB R (G2 R; Supplementary Fig. 3d).

### Impaired antitumor T cell immunity in ICB NR mice

**Fig. 3:**
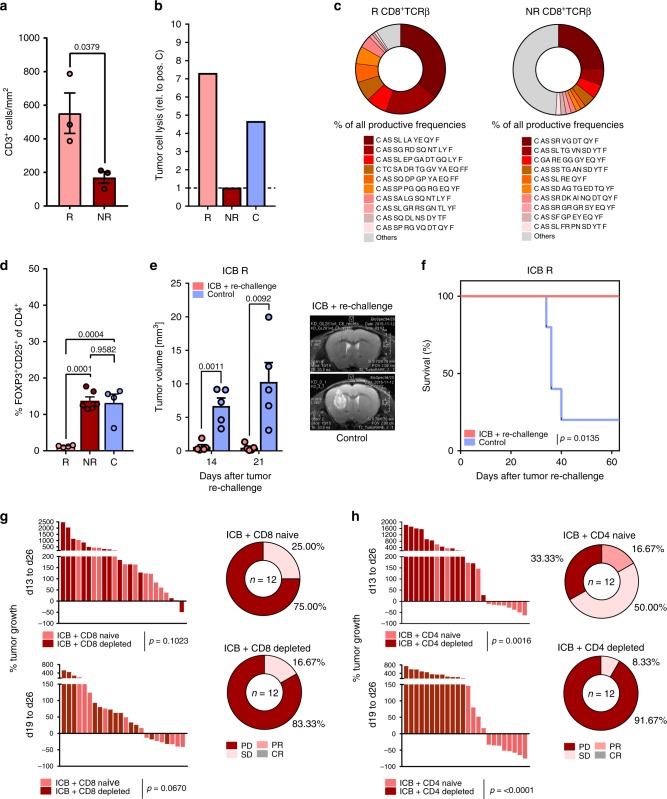
Impaired antitumor T cell immunity in ICB NR tumors. C57Bl/6 J mice were treated with 250 µg anti-PD-1 and 100 µg anti-CTLA-4 (ICB + ), or isotype control (C) on d13, d16, and d19 and tumor monitoring was performed on d13, d19, and d26 post tumor inoculation. **a** CD3^+^ cell counts per mm² tumor area assessed by immunohistochemistry (ICB R *n* = 3, ICB NR *n* = 3 animals). **b** CD3^+^ TILs were isolated by MACS from ICB R, ICB NR, and C tumors on d27 and incubated for 4 h with Gl261 cells ex vivo. Cytotoxicity was analyzed by LDH release relative to positive lysis control (ratio). Five samples per group were pooled. Values are corrected for spontaneous effector and target cell LDH release. **c** Representative ICB R and ICB NR CD8^+^ TCRβ TIL repertoire and % of ten most frequent sequences. **d** Flow cytometry for frequency of CD25^+^FOXP3^+^ T_regs_ of CD4^+^ TILs (ICB R *n* = 4, ICB NR *n* = 6, C *n* = 4 animals). **e**, **f** C57BL/6 J mice were treated with ICB on d14, d17, and d20 after Gl261 injection and tumors were measured on d14, d21, d29, d42, and d50. Gl261 rechallenge of ICB R was performed on d57 after first tumor injection. Tumor volumes on d14 and d21 after rechallenge **e** and survival **f** of Gl261 rechallenged ICB R and control-injected mice (*n* = 5 vs. *n* = 5 animals). **g**, **h** CD8^+^ or CD4^+^ T cells were depleted prior and during ICB using monoclonal depletion antibodies (4 × 500 µg 2.43 or 2 × 1000 µg GK1.5). **g** ICB response in CD8^+^-depleted or naive mice (ICB + CD8 naive *n* = 13, ICB + CD8 depl. *n* = 13 animals) and **h** in CD4 depleted or naive mice (ICB + CD4 naive *n* = 12, ICB + CD4 depl. *n* = 12 animals). Data are represented as mean ± SEM for **a**, **d** and **e**. Statistical significance was determined by one-way ANOVA with Tukey’s test for **d**, two-tailed Student’s *t*-test for **a**, **e**, **g** and **h** or log-rank Mantel–Cox test for **f**. Source data are provided as a Source Data file.

To unravel mechanisms of ICB treatment failure in ICB NR mice, we next examined intratumoral T cell infiltration and T cell cytotoxicity in ICB NR tumors. Although T cell infiltration in ICB NR tumors was significantly lower compared to ICB R tumors (Fig. 3a, Supplementary Fig. 4a), no alterations in relative frequencies of CD8^+^ and CD4^+^ tumor-infiltrating lymphocytes (TILs) of ICB R compared to NR mice were observed (Supplementary Fig. 4a). Antitumor TIL responses of ICB NR TILs were diminished as TILs from ICB NR showed an impaired potency to lyse syngeneic glioma cells ex vivo compared to ICB R and control-treated TILs (Fig. 3b). ICB NR CD8^+^ TILs displayed a more polyclonal T cell receptor (TCRβ) repertoire compared to ICB R CD8^+^ TILs, suggesting a failure of proliferation of tumor-reactive clones in NR tumors (Fig. 3c). This was further supported by the identification of a shared CDR3 sequence motif (alterations of 1 or less AA between ICB R mice) in the CD8 TIL population of ICB R mice that was not present in ICB NR CD8 TILs (Supplementary Fig. 4b).  Additionally, ICB R tumors were characterized by significantly reduced frequencies of regulatory T cells (T_regs_; Fig. 3d). No significant evidence of differential PD-1 surface expression or upregulation of immunosuppressive molecules (CD73 and CD38) on NR CD4^+^ and CD8^+^ TILs was observed (Supplementary Fig. 4d, gating strategy Supplementary Fig. 5a, c). In order to assess if responding mice developed long-term immunity against Gl261 cells, Gl261-bearing mice were treated with ICB as previously described, response was assessed by MRI between d21 and d29, and mice were followed up for 57 days after tumor inoculation until lesions regressed completely or showed stable, minimal lesion volumes. Responding mice were rechallenged with Gl261 cells by intracranial injection into the contralateral hemisphere at day 57 and were followed for 63 days. Here, rechallenged R mice did not develop Gl261 tumors as confirmed by MRI and survival analysis (Fig. 3e, f), suggesting an efficient activation of tumor-reactive T cells and a protective long-term immunity in ICB R mice. Strikingly, depletion of CD8^+^ T cells was not sufficient to abrogate response to ICB, while no tumor showed ICB-induced regression after depletion of CD4^+^ T cells (Fig. 3g, h; Supplementary Fig. 4e-f). This suggests an important role of effector CD4^+^ T cells in driving the response to checkpoint blockade.

### Suppressive myeloid cell infiltrates mediate ICB failure

**Fig. 4:**
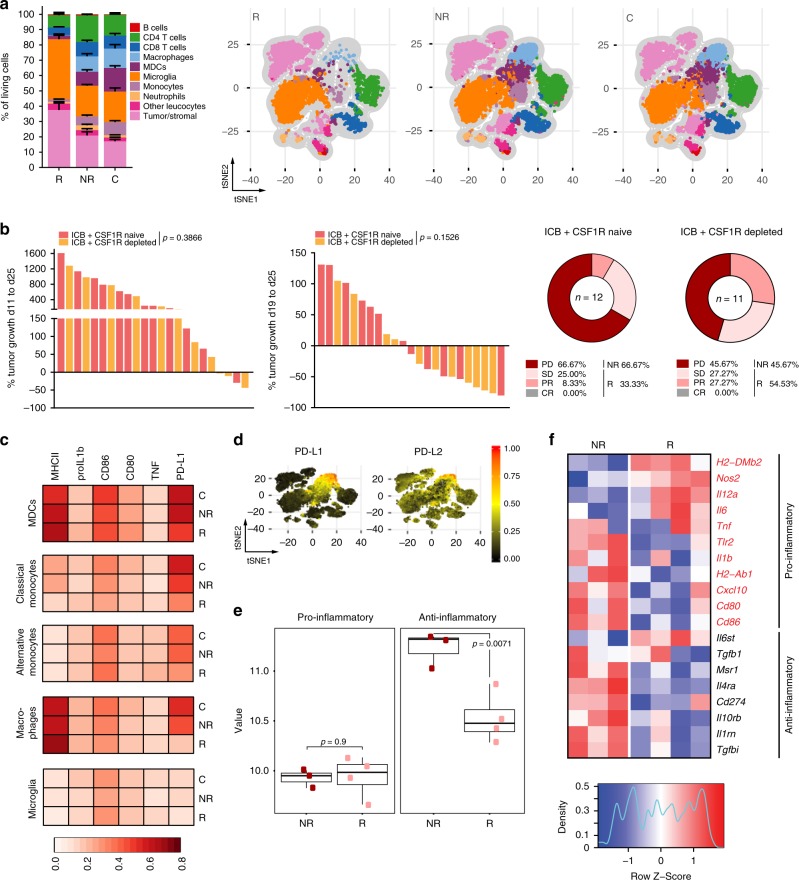
Enhanced frequencies of PD-L1-expressing macrophages in ICB NR tumors. C57Bl/6 J mice were treated with 250 µg anti-PD-1 and 100 µg anti-CTLA-4 (ICB + ), or isotype control (C) on d13, d16, and d19 and tumors were monitored by MRI on d13, d19, and d26 post Gl261 injection. **a** Multiparameter flow cytometry analysis of CNS samples from ICB R, ICB NR, and C on d27. (ICB R *n* = 5, ICB NR *n* = 5, C *n* = 5 animals). *t*SNE-guided immune cell subset identification using *t*SNE composite dimensions by multiparameter flow cytometry analysis. Relative frequencies (left) and FlowSOM-guided meta-clustering on living and single cells (right) of ICB R, NR, and C CNS tissue. **b** CSF1R was targeted prior and during ICB therapy using monoclonal antibodies (AFS98; 6 × 250 µg). Response to ICB therapy in CSF1R-targeted and control mice (ICB + *n* = 12, ICB + CSF1R depleted *n* = 11 animals). **c** Multiparameter flow cytometry analysis of CNS samples from ICB R, ICB NR, and C mice on d27. (ICB R *n* = 5, ICB NR *n* = 5, C *n* = 5 animals). Heatmaps showing the median expression (value range 0–1, white–red) of pro- and anti-inflammatory markers in MDCs, classical monocytes, alternative monocytes, macrophages, and microglia clusters in ICB R, NR, and C CNS tissue. **d** PD-L1 and PD-L2 expression on identified CNS subsets from stochastically selected cells from ICB R, ICB NR, and C CNS tissue. **e**, **f** Pro- and anti-inflammatory gene signature score (geometric mean of pro- and anti-inflammatory genes) **e** and gene expression of pro- and anti-inflammatory genes **f** in tumor-associated CD45^high^CD11b^+^ cells (macrophages) from ICB R and ICB NR assessed by NanoString analysis (ICB R *n* = 4, ICB NR *n* = 3 animals). Center line of the boxplot shows the mean and the whiskers represent the upper and lower most quartiles. Data are represented as mean ± SEM for **a**. Statistical significance was determined by two-tailed Student’s *t*-test for **b** and **e**. Source data are provided as a Source Data file.

Based on the importance of CD4^+^ T cells for mediating response to ICB and their close interaction with myeloid cells, we reasoned that antitumor T cell responses are critically shaped by the intratumoral myeloid compartment interacting with CD4^+^ T cells. Resistance to ICB therapy in other tumor types has previously been linked to tumor-associated macrophages (TAM) and myeloid-derived suppressor cells^17,18^. Therefore, we investigated the presence and phenotype of glioma-infiltrating myeloid cells in ICB R and NR mice. *t*SNE-guided (*t*-Distributed Stochastic Neighbor Embedding) immune cell subset identification by multiparameter flow cytometry analysis revealed markedly decreased frequencies of tumor-infiltrating myeloid cell subsets, including monocytes, monocyte-derived cells (MDCs), and macrophages in ICB R compared with NR animals (Fig. 4a, gating strategy Supplementary Fig. 5a-d, Supplementary Fig. 6a). Of note, there was no evidence for enhanced apoptosis of CD45^high^CD11b^+^ cells in ICB R tumors (Supplementary Fig. 6b, c). Despite decreased frequencies of tumor-infiltrating myeloid cells in ICB R tumors, we did not observe significant differences in the frequency of circulating blood CD11b^+^ cells and their expression of the chemokine receptors CCR2, CCR4, CCR5, and CCR6 involved in myeloid cell recruitment to gliomas during ICB therapy (d15; Supplementary Fig. 6d, e) and response establishment (d21; Supplementary Fig. 6f, g). Moreover, cytokine/chemokine array analysis of ICB R, NR, and control-treated mice did not reveal enhanced plasma levels of myeloid cell-attractant chemokines and factors, such as CCL2, CCL3, CCL4, CCL5, CCL11, CCL17, Macrophage colony-stimulating factor (M-CSF) and granulocyte M-CSF in NR plasma during the early treatment phase (Supplementary Fig. 6h).

Targeting of myeloid cells by CSF1R inhibition has been investigated for the treatment of glioblastoma patients with the aim of (1) hindering myeloid cell infiltration into the tumor and (2) reprogramming suppressive myeloid cells to a pro-inflammatory phenotype^19,20^. Here, we reasoned that CSF1R-targeted therapy might elevate ICB response by releasing T cell suppression by intratumoral myeloid cells. CSF1R blockade by monoclonal antibodies increased therapy response from 33.33% (ICB) to 54.53% (ICB + CSF1R blockade) with only 2 out of 11 mice showing tumor progression of >40% between MRI2 and MRI3 (compared to 6 out of 12 in the ICB cohort; Fig. 4b, Supplementary Fig. 7a, b).

Glioma-associated myeloid cells have been reported to suppress antitumor T cell responses and promote tumor progression^21^. We hence sought to characterize the myeloid cell phenotype, and activation in ICB R and NR tumors in more detail. Intratumoral MDCs and macrophages from ICB R mice expressed higher levels of major histocompatibility complex (MHC) II, while the expression of the immunosuppressive molecules PD-L1 and the poliovirus receptor (CD155) was strongly reduced (Fig. 4c, Supplementary Fig. 8a, b). Expression of PD-L2, the second ligand of PD-1, was not differentially regulated in MDCs and macrophages in ICB R compared to NR tumors (Supplementary Fig. 8b). PD-L1 in the tumor microenvironment was predominantly expressed on intratumoral monocytes, MDCs, and macrophages, while expression of PD-L2 was less restricted and present on other immune cell subsets as well as tumor and stroma cells (Fig. 4d). Moreover, MDCs in ICB R tumors showed increased levels of tumor necrosis factor (Fig. 4c, Supplementary Fig. 8a), suggesting a pro-inflammatory phenotype. In line with these findings, NanoString gene expression analysis of intratumoral CD45^high^CD11b^+^ cells revealed that CD45^high^CD11b^+^ cells from ICB NR tumors displayed an increased expression of anti-inflammatory genes involved in inhibition of IL1b- and IL1a-mediated inflammatory responses (*Il1rn*), IL4 signaling (*IL4ra*), and M2-associated genes, such as *PD-L1*, *TGFbi*, and the scavenger receptor *Msr1* (Fig. 4e, f). Contrary, CD45^high^CD11b^+^ cells from ICB R tumors upregulated pro-inflammatory genes known to induce Th1 T cell responses, including the cytokine *Il12* and genes involved in MHC II presentation (*H2-DMb2*). As PD-L1 was differently expressed on the majority of myeloid cell populations with the strongest differences on intratumoral macrophages (Fig. 4c, Supplementary Fig. 8a), we sought to investigate the impact of PD-L1 expression on the effector functions of ICB R and NR tumor-associated myeloid cells. Interestingly, differences in PD-L1 expression between ICB R and NR were exclusively observed in myeloid cells in the tumor microenvironment and not present in the periphery (Supplementary Fig. 8c). Strikingly, PD-L1 expression on intratumoral CD11b^+^ myeloid cells showed a strong negative correlation with response to ICB therapy (Supplementary Fig. 9a). The immunosuppressive molecule PD-L1 is known to inhibit T cell proliferation and effector function upon binding to its ligand PD-1 on T cells^1^. As PD-1 was blocked in ICB-treated mice, we investigated whether PD-L1/PD-1 signaling directly impacts macrophage activation and function, such as phagocytosis as previously proposed^22,23^. Here, we did not observe an increased phagocytotic activity of tumor-associated CD45^high^CD11b^+^ cells isolated from ICB R compared to ICB NR mice (Supplementary Fig. 9b). Moreover, ex vivo PD-L1 inhibition during phagocytosis did not induce enhanced phagocytic activity of TAM (Supplementary Fig. 9b). Notably, CD11b^+^ PD-L1^−^ cells in the tumor microenvironment of ICB R mice showed increased expression of MHC II compared to NR PD-L1^−^ myeloid cells, suggesting enhanced antigen presentation by this PD-L1^−^ myeloid cell subset (Supplementary Fig. 9c). To rule out that increased frequencies of suppressive myeloid cells in ICB NR tumors are a result of increased tumor size independent of treatment rather than a mechanism of ICB resistance, we correlated MRI3 tumor volumes with frequencies of tumor-associated CD45^high^CD11b^+^ cells and percentage of PD-L1^+^ cells on tumor-associated CD45^high^CD11b^+^ cells in ICB and C-treated mice. While MRI3 tumor volumes strongly correlated with both factors in ICB mice, no correlation was observed in C mice (Supplementary Fig. 10a–c, left panel). Moreover, frequencies of tumor-associated CD45^high^CD11b^+^ cells and percentage of PD-L1^+^ cells on tumor-associated CD45^high^CD11b^+^ cells were significantly correlated with tumor growth (MRI1 to MRI3, and MRI2 to MRI3) in ICB but not C mice (Supplementary Fig. 10a–c, middle (MRI1 to MRI3) and right (MRI2 to MRI3) panel). Heterogeneity of tumor volumes in C mice does not reflect a heterogeneity in the suppressive CD11b compartment.

### CD4^+^ TIL suppression by the PD-L1/PD-1/CD80 axis

**Fig. 5:**
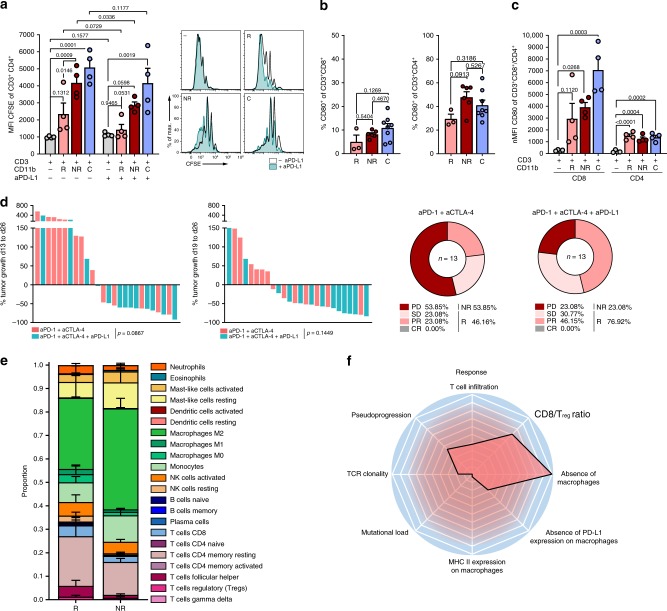
TAMs establish ICB resistance through PD-L1-CD80-mediated CD4^+^ T cell suppression and T_reg_ expansion. **a**, **c** Ex vivo T cell suppression by tumor-associated myeloid cells. CD11b^+^ cells were purified from ICB R, ICB NR, and C tumors on d27 by MACS and co-cultured for 72 h with pre-activated naive CD3^+^ splenocytes with and without 20 µg ml^−1^ anti-PD-L1 (10 F.9G2; ICB R *n* = 4, ICB NR *n* = 4, C *n* = 4 animals). **a** CD4^+^ T cell proliferation after co-culture assessed by CFSE staining. **b** Frequency of CD80^+^ cells of CD8^+^ (left) and CD4^+^ (right) TILs (ICB R *n* = 3, ICB NR *n* = 6, C *n* = 8 animals). **c** CD80 expression on pre-activated naive CD4^+^ and CD8^+^ T cells before and after co-culture with tumor-associated myeloid cells from ICB R, ICB NR, and C. **d** Tumor growth (left) and response (right) of C57BL/6 J mice treated with 250 µg anti-PD-1 and 100 µg anti-CTLA-4, and as combinatory therapy with additional 200 µg anti-PD-L1 on d13, d16, and d19 post Gl261 inoculation (aPD-1 + aCTLA-4 *n* = 13, aPD-1 + aCTLA-4 + aPD-L1 *n* = 13 animals). **e** CIBERSORT analysis of a GBM expression dataset of PD-1 inhibitor-treated patients before therapy (R *n* = 4, NR *n* = 5 biologically independent samples)—two-sided WRST. **f** Mediators of ICB response (*Z*-transformed log2 fold change R/NR). Data are represented as mean ± SEM for **a**, **b**, **c** and **e**. For **a**, statistical significance was determined by one-way ANOVA in combination with Dunnett’s test (CD3^+^ cells + R, NR, or C CD11b^+^ cells vs. T cells only) and Sidak’s test for multiple comparison (CD3^+^ cells + R CD11b^+^ cells vs. CD3^+^ cells + NR CD11b^+^ cells) or two-tailed paired Student’s *t*-test (− PD-L1 vs. + PD-L1). Statistical significance was analyzed by one-way ANOVA with Tukey’s test for multiple comparison for **b**, by one-way ANOVA in combination with Dunnett’s test (CD3^+^ cells + R, NR, or C CD11b^+^ cells vs. T cells only) for **c**, by unpaired two-tailed Student’s *t*-test for **d**, and WRST with Benjamini–Hochberg correction for **e**. Source data are provided as a Source Data file.

To address whether of PD-L1-expressing myeloid cells directly impact T cell activation and proliferation in the tumor microenvironment, we analyzed the ability of macrophages isolated from ICB R, NR, and control tumors to suppress T cell proliferation and effector function. Suppression of CD4^+^, but not CD8^+^ T cell proliferation was more pronounced when T cells were co-cultured with tumor-associated myeloid cells from ICB NR compared to R (Fig. 5a, Supplementary Fig. 11a–c). This was accompanied by an increased expansion of T_regs_ after co-culture with tumor-associated myeloid cells from ICB NR (Supplementary Fig. 11d). Furthermore, CD4^+^ T cell suppression and T_reg_ expansion were reduced upon PD-L1 inhibition during co-culture with ICB NR tumor-associated myeloid cells (Fig. 5a, Supplementary Fig. 11c, d). As PD-1 is blocked by the ICB regimen used here, we hypothesized that T cell suppression is established by an alternative binding partner of PD-L1 on T cells. Interestingly, CD80 has been proposed to act as an alternative binding partner of PD-L1 on T cells, thereby suppressing T cell proliferation and activation^24^. Indeed, we confirmed CD80 expression on CD4^+^ and CD8^+^ T cells of ICB-treated mice, with a predominant expression in the TIL compartment (Fig. 5b, Supplementary Fig. 11e). Moreover, CD80 expression on naive, pre-activated T cells was induced upon co-culture with tumor-associated myeloid cells and levels of CD80 positive CD4 T cells after co-culture with tumor-associated myeloid cells was comparable to levels on CD4 TILs (Fig. 5c, Supplementary Fig. 11e). To investigate if blockade of the PD-L1/CD80 interaction can restore response to anti-PD-1 + anti-CTLA-4 therapy, PD-L1 blocking antibodies were administered in addition to the anti-PD-1 and anti-CTLA-4 regimen. Triple ICB resulted in a decreased tumor growth and enhanced response (11/13 vs. 6/13) to ICB therapy when compared with ICB targeting PD-1 and CTLA-4 only (Fig. 5d, Supplementary Fig. 11f). To confirm these preclinical findings, we evaluated macrophage frequencies in glioblastoma patients from a recently published clinical trial of anti-PD-1 treatment^25^. From this dataset, we applied CIBERSORT analysis of RNA sequencing data from GBM tissue of R (*n* = 4) and NR (*n* = 5) patients before anti-PD-1 therapy. In line with our findings, intratumoral M2 macrophage levels as well as myeloid cell infiltrate levels (monocytes, M0, M1, and M2 macrophages) showed a trend toward elevated levels in NR glioblastoma patients before anti-PD-1 therapy (Fig. 5e), supporting the hypothesis that suppressive myeloid cell subsets impair the induction of antitumor T cell responses by ICB therapy. In summary, our findings suggest a distinct set of biomarkers associated with response to ICB in a hypermutated syngeneic glioma model that is dominated by innate (absence of intratumoral macrophages and absence of PD-L1 on intratumoral macrophages) rather than adaptive immune parameters (Fig. 5f).

## Discussion

Immune checkpoint inhibitors for glioma patients are now tested in clinical trials and first results point toward poor responses, although neo-adjuvant ICB therapy was recently shown to promote a survival benefit in glioblastoma patients^6,26,27^. These studies indicate that mechanism-driven response biomarkers and combination strategies are required, in order to define which patients benefit from checkpoint therapy and to simultaneously enhance therapy response.

Preclinical models have been notoriously problematic for the identification of biomarkers, not only as they incompletely reflect tumor biology but also because they seemingly lack interindividual heterogeneity. We have uncovered and mechanistically dissected the surprising finding that heterogeneity of response is not only observed in humans but also in syngeneic tumor models in inbred mice. This observation may offer the opportunity to not only elucidate novel mechanisms of response and resistance and, novel therapeutic targets suitable for combination therapies, but also identify potential predictive biomarkers potentially applicable to patients with gliomas (Fig. 5f). We have utilized this robust heterogeneity of response and resistance with pseudoprogression, signifying an immune response in a syngeneic high-mutational load experimental glioma model to establish a radiomic-based MRI signature, predicting response with high accuracy. This signature may be useful for future clinical trials enriched for patients with hypermutated glioblastoma similar to the experimental model used here. Proposed signature might additionally be applied in combination with automated quantitative tumor response assessment of MRI, using artificial networks that will allow for improved clinical decision making^28^. The establishment and application of additional MR protocols to image immunotherapy-induced immune responses in the CNS will further facilitate therapy monitoring and response evaluation.

Heterogeneity of response allowed for the precise analysis of the glioma immune microenvironment associated with response and resistance in this model. We identified a PD-L1^+^ macrophage subset that drives resistance to ICB by suppression of CD4^+^ T cell activation and proliferation and T_reg_ induction (Fig. 5a, Supplementary Fig. 11c, d). PD-L1 expression of tumors has long been believed to be the major prerequisite for efficient PD-1 blockade. High PD-L1 expression in the initial tumor tissue was associated with poor response to nivolumab in the CheckMate 143 trial (NCT02017717) for patients with recurrent glioblastoma^6^. Loss of *PTEN* has been associated with an increase of PD-L1 on glioblastoma cells and immune resistance^29–31^ and in clinical trials resistance to PD-1 inhibitors is associated with genetic alterations in the *PTEN* gene^25^. However, PD-L1 is not only expressed on tumor cells but also infiltrating leukocytes in glioblastoma^32–34^ and PD-L1 expression on macrophages has been associated with poor survival and resistance to immunotherapy for patients with glioblastoma^35,36^. Recent studies highlight the impact of tumor-derived factors on PD-L1 expression on macrophages^37–39^. These clinical studies and our data are in line with previous observations that macrophage infiltration and PD-L1 expression on infiltrating macrophages are critical determinants of resistance to ICB. It is a widely accepted view that signaling induced by PD-1/PD-L1 interaction impairs T cell effector function and proliferation that subsequently results in T cell exhaustion and decreased tumor immunity^1^. However, the impact of PD-1/PD-L1 binding on the phenotype, and function of PD-L1^+^ antigen-presenting cells and tumor cells is still incompletely understood. Here, we have shown that PD-L1 expression on macrophages is accompanied by the expression of other immunosuppressive molecules such as CD155 (Supplementary Fig. 8b) and that frequency of PD-L1^+^ myeloid cells is negatively correlated to therapy response to checkpoint blockade (Supplementary Fig. 9a). This data is supported by evidence that suggests an induction of a regulatory macrophage profile of PD-L1^+^ macrophages upon PD-L1 signaling^40^. Binding of PD-1 on T cells to PD-L1 on macrophages hence decreases inflammatory mediators while increasing the production of anti-inflammatory cytokines^40^. It remains to be investigated, if this also holds true for the CD80/PD-L1 interaction. We further hypothesized that PD-1/PD-L1 signaling might interfere with macrophage effector function, such as phagocytosis. Interestingly, PD-1 expression on TAMs has been reported to increase during tumor progression in murine and human tumors, and to negatively correlate with phagocytic potency against tumor cells^22^. In our hands, glioma-associated macrophages did not induce phagocytosis upon PD-L1 inhibition, suggesting an alternative mechanism of immunosuppression by PD-L1^+^ macrophages. Here, we show that combination of PD-1, CTLA-4, and PD-L1 enhances response rates and that PD-L1^+^ macrophages suppress T cell proliferation under PD-1 and CTLA-4 blockade by a compensatory mechanism through CD80 binding.

As differences in PD-L1 expression were exclusively observed on intratumoral macrophages and were not present in the periphery, additional biomarkers will be required to monitor therapy response to ICB, especially in the context of glioblastoma patients. These biomarkers might address soluble factors of macrophage recruitment, polarization, and PD-L1-inducing factors in the blood. It moreover remains to be investigated if macrophage-mediated resistance to ICB is acquired or preexisting, as a preexisting mechanism might be exploited to stratify and select patients that benefit from checkpoint blockade therapy using tumor samples.

In conclusion, this evidence suggests an important role of intratumoral macrophages-expressing PD-L1 and other immunosuppressing molecules in the response to PD-1 and CTLA-4 blockade, thereby inhibiting the induction of proliferation and reactivation of tumor-reactive T cells. Strategies to enhance therapy response to ICB might thus involve the mechanism-driven combination of ICB and targeting of TAMs.

## Methods

### Mice

C57Bl/6 J wild-type mice were purchased from Charles River or Janvier Laboratories at the age of 6–8 weeks. Animal procedures were performed in the accordance with all relevant ethical regulations for animal testing and research, and were approved by the governmental authorities (Regional Administrative Authority Karlsruhe, Germany). Sex- and age-matched mice were used for further experiments. If not stated otherwise, female mice were used for the experiments. All mice were 7–12 weeks of age at use. Mice were kept under specific-pathogen-free (SPF) conditions at the animal facility of the DKFZ Heidelberg.

### Cell culture

Gl261 cells were purchased from the National Cancer Institute. Gl261 cells were cultured in Dulbecco’s modified Eagle’s medium (DMEM) supplemented with 10 % fetal bovine serum (FBS), 100 U ml^−1^ penicillin, and 100 µg ml^−1^ streptomycin (Sigma-Aldrich) at 37 °C, 5% CO_2_. Gl261 cells were routinely tested for viral, mycoplasma, and non-murine cell contamination by multiplex cell contamination test (Multiplexion GmbH)^41^. Primary murine T cells and myeloid cells were cultured in RPMI-1640 (Sigma-Aldrich) with 10% FBS, 100 U ml^−1^ penicillin, 100 μg ml^−1^ streptomycin, 25 mM Hepes pH 7.4, 1 mM sodium pyruvate, 5 × 10^−5^ M 2-mercaptoethanol (Sigma-Aldrich), and 2 mM L-glutamine (Thermo Fisher) at 37 °C, 5% CO_2_.

### Gl261 tumor cell inoculation and tumor rechallenge

A total of 1 × 10^5^ Gl261 tumor cells were diluted in 2 µl sterile phosphate-buffered saline (PBS; Sigma-Aldrich) and stereotactically implanted into the right hemisphere of 7–9-week-old female C57Bl/6 J mice (coordinates: 2 mm right lateral of the bregma and 1 mm anterior to the coronal suture with an injection depth of 3 mm below the dural surface), using a 10 µl Hamilton micro-syringe driven by a fine step stereotactic device (Stoelting). Tumor cell inoculation was performed under anesthesia and mice received analgesics for 2 days post operation. Mice were checked daily for tumor-related symptoms and sacrificed when tumor burden and stop criteria were met or mice showed signs of neurological deficit. For tumor rechallenge experiments 7–9-week-old male C57Bl/6 J mice (Charles River) were intracranially injected with 100.000 Gl261 cells and mice were treated with anti-PD-1 + anti-CTLA-4 on day 14,17, and 20 post inoculation. Tumor growth was monitored by MRI on day 14, 21, 29, 42, 70, and 78 post inoculation. Responding mice were rechallenged with 1 × 10^5^ Gl261 cells by intracranial injection into the contralateral hemisphere on day 57 post inoculation, as described above. In addition, 1 × 10^5^ Gl261 cells were injected into a control group of five naive, age and sex-matched C57Bl/6 J mice. Mice were checked daily for tumor-related symptoms and sacrificed when tumor burden and stop criteria were met or mice showed signs of neurological deficit. Mice were followed for 63 days post tumor rechallenge (120 days after the first tumor injection) and survival was analyzed by Kaplan–Meier survival curves using log-rank Mantel–Cox test.

### In vivo antibodies

For immune checkpoint therapy, 100 µg anti-CTLA-4 (9D9, BioXCell) per mouse and 250 µg anti-PD-1 (RMP1-14, BioXCell) per mouse or equivalent doses of isotype control antibodies (MCP-11 and 2A3, BioXCell) were administered by intraperitoneal (i.p.) injection in 200 µl PBS on day 13, 16, and 19 after tumor inoculation. PD-L1 blockade (200 µg per mouse 10 F.9G2 or LTF-2 isotype control, BioXCell) was performed by i.p. injection in combination with anti-PD-1 and anti-CTLA-4 therapy on day 13, 16, and 19 after tumor inoculation. For PD-1 monotherapy, C57Bl/6 J mice were treated with 250 µg anti-PD-1 or isotype control (C) on day 10, 13, and 16 and tumor growth was monitored by MR imaging on day 10, 17, and 24 post intracranial Gl261 tumor injection. For CD4 T cell blockade, 1000 µg GK1.5 or LTF-2 isotype antibody per mouse were administered by i.p. injection on day 13 and 20 after tumor injection. CD8 T cell blockade was performed using 500 µg anti-CD8 2.43 or LTF-2 isotype antibody per mouse on day 13, 17, 20, and 24 after tumor inoculation. In CD4 and CD8 blocking experiments, ICB with anti-PD-1 and anti-CTLA-4 was performed on day 15, 18, and 21 post inoculation to allow for T cell depletion before therapy start. Efficacy of CD4 and CD8 depletion was confirmed before and during immune checkpoint therapy (every third day) by flow cytometry analysis of peripheral blood lymphocytes and by terminal flow cytometry analysis of TILs. For CSF1R blockade, 250 µg AFS98 or 2A3 isotype antibody per mouse were administered by i.p. injection on day 11, 14, 17, 20, 23, and 25 after tumor injection. ICB with anti-PD-1 and anti-CTLA-4 in combination with CSF1R depletion was performed on day 13, 16, and 19 post inoculation.

### Survival experiments

For survival experiments, 1 × 10^5^ Gl261 tumor cells were implanted into the right hemisphere of 7–9-week-old female C57Bl/6 J mice as described above. Mice were treated with anti-PD-1 + anti-CTLA-4 or isotype control on day 13, 16, and 19 post inoculation and MRI was performed on day 13, 19, and 26 as described above. Mice were checked daily for tumor-related symptoms and sacrificed when tumor burden and stop criteria were met or mice showed signs of neurological deficit. For survival data, data of two independent experiments were combined.

### Tumor imaging and response criteria

MRI of Gl261 tumors was performed on day 13, 19, and 26 post tumor inoculation on a 9.4 Tesla horizontal bore small animal NMR scanner (BioSpec 94/20 USR, Bruker BioSpin GmbH) with a four-channel phased-array surface receiver coil. MRI was performed under inhalation anesthesia with isoflurane. On day 13 post inoculation, mice were grouped according to tumor size. Tumor volumes and diameters were retrieved from standard T2-weighted sequences (TE: 33 ms; TR: 2500 ms) and tumor volume was manually segmented in the Osirix or ITKsnap imaging software in a blinded fashion regarding treatment condition. Treatment response was assessed analogous to the clinically established Immunotherapy Response Assessment in Neuro-Oncology (iRANO) criteria^15^. Specifically, CR was defined as relative increase in lesion volume MRI1–MRI3 (%*V*_MRI3–MRI1_) of −100%, PR as %*V*_MRI3–MRI1_ ≤ −65.0% and/or %*V*_MRI3–MRI2_ ≤ −65.0%, SD as %*V*_MRI3–MRI1_ > −65% and < + 40%, and PD as %*V*_MRI3–MRI1_ ≥ + 40%. Mice with unconfirmed progression between MRI2 and MRI3 were defined as SD, if tumors regressed at least 30% between MRI2 and MRI3 (%*V*_MRI3–MRI2_ ≤ −30.0%). Criteria for PD (NR) were met if tumor volume increased by >=40% between MRI1 and MRI3 (thereby corresponding the 25% increase in the biperpendicular diameter mandated by the iRANO criteria, assuming spherical configuration of the tumor). Mice with CR, PR, or SD were defined as R mice. For validation of T2-weighted MR-based response evaluation, T2-w imaging data of ICB- and control-treated mice was compared to T1-w monitoring (T1-w parameters: after iv administration of 0.01 mmol Gadoteric acid: RARE, coronal aquisition, matrix size 200 × 200, TE 6 ms, TR 1000 ms, two averages, flip angle 90°, refocusing angle 180°, resolution; 100 μm × 100μm, slice thickness 0.7 mm).

### Radiomic signature discovery and response prediction

Radiomic analysis of MRI data was performed with an established workflow as described previously^42,43^. Briefly, radiomic features were calculated from the T2-hyperintense tumor volume from the first MRI and the change in features between the first and second MRI for radiomic signature discovery (Supplementary Data 1). Based on these radiomic features (*n* = 423 from each time point) gradient boosting machine-learning models were constructed to predict treatment failure at the third MRI. Model performance was evaluated using fivefold cross validation. In more detail, lesion volumes (MRI1 baseline lesion volumes, MRI2 during treatment lesion volumes, and MRI3 post treatment lesion volumes) were segmented on T2-weighted MR imaging using a region-growing segmentation algorithm implemented in ITK-SNAP (www.itksnap.org). Radiomic features were calculated from these tumor segmentation masks from T2-weighted MR imaging for each mouse from both time points using the medical imaging interaction toolkit (MITK, www.mitk.org)^44^. This included (i) 146 first-order features (ii) 33 volume and shape features, (iii) 200 texture features, and 44 curvature features (CF). Next, a radiomic feature set consisting of all features from MRI1 as well as the absolute difference in each radiomic feature between time points MRI1 and MRI2 was used as an input for predictive modeling of treatment failure (i.e., prediction of response yes vs. no) at MRI3 (implemented using R version 3.5.1 (R Foundation for Statistical Computing, Vienna, Austria) with the caret library^45^). All radiomic features were *z*-score normalized (i.e., transformed to a mean of 0 and a standard deviation equal to 1). Predictive modeling was performed using a gradient boosting machine-learning algorithm that iteratively constructs an ensemble of weak decision tree learners through boosting to form a single strong predictive model (the tuning parameters (boosting iterations, max tree depth, shrinkage, and min. terminal node size) were automatically optimized via resampling procedures). The performance of the gradient boosting classifier was assessed based on a two-times repeated fivefold cross validation resampling procedure. The held-out predictions in each of the resampling iterations were used to calculate the accuracy, area under the receiver operating charasteristic (ROC), sensitivity, specificity, no information rate (largest class percentage for each molecular parameter, i.e., the prediction or accuracy by chance), and a hypothesis test (using the binom.test function) to evaluate whether the accuracy rate is greater than the no information rate. *P* < 0.05 were considered significant.

### Mutanome analysis Gl261 tumors

DNA from Gl261 tumor tissue from R and NR mice was extracted using the INVISORB^®^ DNA Tissue Mini Kit (STRATEC Biomedical AG) according to the manufacturer’s instruction. RNA contamination was eliminated by RNase digestion with 10 mg ml^−1^ RNase at room temperature (RT) for 5 min (Sigma-Aldrich). Exome sequencing was performed on the Illumina NextSeq500 platform (Illumina Inc, San Diego, Calif.) using High output flow cell (75 nt reads paired end + 8 nt index). SureSelectXT Target Enrichment System (Agilent Technologies) was used for library generation according to the manufacturer’s instructions. To convert the vendor-specific sequencing data format generated by the Illumina NextSeq500 to a standard file format, the Illumina tool bcl2fastq (v2.15.0.4)^46^ was used. To check the sequencing read quality, reports were generated with the tool fastqc (v0.10.1)^47^. After quality checks the alignment was performed with bwa mem (v0.7.5)^48^ and the mouse reference genome GRCm38.68. The picard-tools (v1.105)^49^ were used to remove duplicates from the alignment files. The sorting and indexing of these files was done with samtools (v0.1.19)^50^. Afterward the variants were called by samtools mpileup (v0.1.19) for single-nucleotide variants and platypus (v0.7.9.1)^51^ for insertions and deletions. The basic annotations of the called variants was done with annovar (v2013-08-23)^52^.

### B16 tumor experiments

B16 melanoma cells were kindly provided by Günther J. Hämmerling (Division of Molecular Immunology, DKFZ Heidelberg). B16 cells were cultured in DMEM supplemented with 10 % FBS, 100 U ml^−1^ penicillin, and 100 µg ml^−1^ streptomycin (Sigma-Aldrich) at 37 °C, 5% CO_2_. B16 cells were routinely tested for viral, mycoplasma, and non-murine cell contamination by multiplex cell contamination test (Multiplexion GmbH)^41^. For B16 tumor cell inoculation, cell suspension in PBS was mixed with an equal volume of Matrigel® Basement Membrane Matrix (Corning®) and 5 × 10^4^ cells in 200 µl of cell-matrix suspension were injected subcutaneously into the right flank of C57BL/6 J mice. Tumor growth was monitored by two-dimensional measurements using a caliper (area: width × length). A total of 100 µg per mouse anti-CTLA-4 (9D9, BioXCell) and 250 µg per mouse anti-PD-1 (RMP1-14, BioXCell) or equivalent doses of isotype control antibodies (MCP-11 and 2A3, BioXCell) were i.p. injected in 200 µl PBS on day 7, 10, and 13 after tumor inoculation. Flow cytometry analysis of tumor-infiltrating and peripheral immune cells was performed on day 15 post inoculation.

### CD3 Immunohistochemistry

Mice were sacrificed by cardial perfusion with PBS, excised brains were embedded in Tissue-Tek® O.C.T.TM (Sakura), and snap-frozen in cold 2-methylbutane (Sigma-Aldrich) on dry ice. Fresh-frozen sections were stained for CD3 with 1:100 rabbit anti-human/mouse CD3 (Dako; A 0452). In brief, cryo-sections were fixed with 4.5% paraformaldehyde and quenching of endogenous peroxidase was performed with 0.3% H_2_O_2_. Sections were further washed and blocked with 4% normal goat serum in PBS at RT for 1 h. CD3 was stained at 4 °C over night and secondary antibody incubation was performed with biotinylated goat anti-rabbit IgG (1:200; Vector; BA-1000) in 4% normal goat serum for 45 min at RT. After washing with PBS, the VECTASTAIN Elite ABC HRP Kit (Vector) was applied for 30 min at RT. Slides were washed with PBS and developed with 3,3′-Diaminobenzidine (DAB; Dako;). Reaction was stopped with dH_2_O. Cryo-sections were further counterstained with hematoxylin for 3 min at RT and developed in tap water for 10 min. Tissue sections were washed with dH_2_O followed by dehydration with 70% EtOH, 96% EtOH, and 100% EtOH. Slides were cleared thrice with Histo-Clear at RT for 3 min and mounted with histomount medium. Images were acquired on Zeiss Cell Observer using the ZEN software. Quantitative analysis of CD3^+^ T cell numbers per mm² tumor area was performed with ImageJ.

### TCR sequencing and GLIPH analysis

DNA from TIL samples was extracted by QIAamp DNA Micro Kit (Qiagen; 56304) according to the manufacturer’s instruction. TCRβ sequencing was performed using the TCRβ CDR3 Adaptive Biotechnologies® sequencing technology (immunoSEQTM Kit; Adaptive Biotechnologies; Seattle; WA)^53,54^. Samples were sequenced on the Illumina NextSeq500 platform (Illumina Inc, San Diego, Calif.) using MID output flow cell (156nt reads + 15nt Index). Data were analyzed with the ImmunoSEQ analyzer toolset and presented as productive amino acid sequences. Clonality was assessed by the percentage of the top ten frequent clones of all identified productive sequences, or productive clonality. Sequence similarity analysis was performed using R GLIPH analysis as adapted from Glanville et al.^55^. TCR sequences from healthy spleen and thymus from C57BL/6 J mice were used as reference database.

### Processing of spleen, blood and tumor tissue

Spleens were excised and meshed twice through a 70 μm cell strainer to obtain a single-cell suspension and erythrocytes were lysed with ACK buffer containing 150 mM NH_4_Cl, 10 mM KHCO_3_, and 100 μM Na_2_EDTA. Blood samples were obtained by submandibular vein (immune cell monitoring during experiments) or cardial puncture in deep anesthesia (terminal immune cell analysis) and collected in syringes or tubes coated with 0.5 M EDTA. Erythrocytes were lysed with ACK buffer and cells were washed twice and further processed for flow cytometry analysis. For isolation of TILs, mice were cardially perfused in deep anesthesia. For Gl261 tumors, the right hemisphere was excised and the cerebellum removed. For B16 tumors, flank tumors were excised. B16 tumors and Gl261-bearing hemispheres were mechanically dissected and enzymatically digested in HBSS (Sigma-Aldrich, 11088866001) supplemented with 50 µg ml^−1^ Liberase DL (Roche) under slow rotation at 37 °C for 30 min. Cells were subsequently meshed through a 100 μm and 70 µm cell strainer, stained, and analyzed by flow cytometry. For Gl261 cell suspensions, cells were purified using myelin removal beads II (Miltenyi Biotec; 130-096) according to the manufacturer’s instruction.

### Flow cytometry

For intracellular cytokine staining, cells were incubated with 5 µg ml^−1^ Brefeldin A (Sigma-Aldrich) for 5 h at 37 °C, 5% CO_2_ to allow for intracellular enrichment of cytokines. Brain tumor and spleen cell suspensions were blocked with anti-CD16/CD32 (eBioscience; 93; 14-0161) and extracellular targets were stained at 4 °C for 30 min (Supplementary Data 2). Intracellular antigens were fixed, permeabilized, and stained using the FOXP3/transcription factor staining buffer set (eBioscience; 00-5523) and the antibodies listed in Supplementary Data 2. Staining of intracellular targets was performed for 45 min at 4 °C. Stained lymphocytes were analyzed on FACS Canto II (BD Biosciences; Germany) or on Attune NxT (Thermo Fisher; Germany). FlowJo V9 or V10 were used for data analysis. Multiparameter FACS data were generated on a FACSSymphony (BD Biosciences) using the antibodies described in Supplementary Data 2. Data were compensated, exported (FlowJo V10), uploaded, and normalized using Cyt3 (Matlab_R2018b). The new generated FCS files were uploaded in Rstudio (Version 1.1.463). *t*SNE (displaying stochastically selected events from all different conditions) and FlowSOM (events from each condition) were performed as described by Brumelman et al.^56^.

### Ex vivo phagocytosis

For isolation of CD11b^+^ cells of Gl261 tumors from ICB R, ICB NR, and C mice, myelin was removed of tumor single-cell suspension with myelin removal beads II (Miltenyi Biotec; 130-096) according to the manufacturer’s instruction. Subsequently, CD11b^+^ cells were purified using MagniSort™ Mouse CD11b Positive Selection Kit (eBioscience; 8802-6860-74). Ex vivo phagocytosis of CD11b^+^ cells was assessed as previously described^22^. In brief, CD11b^+^ cells were plated onto ultra-low attachment 96-well plates (Corning) and incubated at 37 °C, 5% CO_2_ for 20 min to allow for cell resting. CD11b^+^ cells were subsequently cultured at 37 °C, 5% CO_2_ for 2 h with pHrodo™-red *Staphylococcus* *aureus* BioParticles (Thermo Fisher) according to the manufacturer’s instruction. Phagocytosis was assessed by flow cytometry analysis for pHrodo-red^+^ cells of macrophages (CD45^high^CD11b^+^ cells) and microglia (CD45^low^CD11b^+^ cells). PD-L1 was blocked during incubation with pHrodo™-red *S*. *aureus* BioParticles with 20 µg ml^−1^ anti-PD-L1 (10 F.9G2; BioXCell).

### Apoptosis of intratumoral macrophages

Macrophages (CD45^high^CD11b^+^) of ICB R and ICB NR were stained with annexin V-FITC (BioVision, 1:100) and DAPI (1:250, Invitrogen, Carlsbad, USA) in Annexin V binding buffer (eBioscience, Germany) at RT for 15 min, and analyzed using BD-FACS Canto II. Early apoptosis was defined by single annexin V positivity. Late apoptosis was defined as annexin V and DAPI double positivity.

### Blood immune cell monitoring

Blood samples of ICB-treated mice were collected on day 15 and 21 after Gl261 inoculation using submandibular vein puncture and collected in tubes coated with 0.5 M EDTA. Erythrocytes were lysed with ACK buffer containing 150 mM NH_4_Cl, 10 mM KHCO_3_, and 100 μM Na_2_EDTA. Cells were washed twice with PBS and further processed for flow cytometry analysis.

### Plasma cytokine array

Blood samples were collected by submandibular vein puncture and plasma was obtained by centrifugation at 2000×*g*, RT for 10 min. Plasma cytokine analysis was performed with pooled plasma samples at equal ratios for five mice per group according to the manufacturer’s instructions (Proteome Profiler™ Array Mouse Cytokine Array Panel A; R&D Systems; ARY006). Samples were measured on the ChemiDocTM MP Blot reader system (BioRad; Hercules, Calif.). ImageJ 1.48 and the Gilles Carpentier’s Protein Array Analyzer for ImageJ toolset were used for data analysis.

### Ex vivo and in vitro T cell suppression

For ex vivo and in vitro T cell suppression assays, T cells were purified from spleens of naive C57BL/6 J mice using the MagniSort™ Mouse T cell Enrichment Kit (eBioscience; 8802-6820), labeled with 5 µM carboxyfluorescein succinimidyl ester (CFSE; Thermo Fisher; C34570) and pre-activated prior to myeloid cell co-culture with plate-bound 0.1 µg ml^−1^ anti-CD3 (145-2C11; eBioscience;) and 1 µg ml^−1^ anti-CD28 (37.51; Biolegend) at 37 °C, 5% CO_2_ for 16–18 h. Ex vivo T cell suppression assay with tumor-associated myeloid cells was adapted from De Henau et al.^18^. In brief, Gl261-associated myeloid cells were isolated of from ICB R, ICB NR, and C mice. To this end, single-cell suspensions of tumor-bearing hemispheres were subjected to myelin removal (Myelin removal beads II; Miltenyi Biotec; 130-096) and CD11b^+^ cells were purified by MACS using the MagniSort™ Mouse CD11b Positive Selection Kit (eBioscience; 8802-6860). Gl261-associated CD11b^+^ cells were co-cultured with pre-activated T cells at a ratio of 1:1 (2.5 × 10^4^ CD3^+^T cells and 2.5 × 10^4^ CD11b^+^ myeloid cells) in murine T cell proliferation medium at 37 °C, 5% CO_2_ for 72 h. T cell proliferation was examined by CFSE mean fluorescence intensity of living CD3^+^ CD8^+^ and living CD3^+^ CD4^+^ T cells, and percentage of cells per cell division.

### Ex vivo TIL cytotoxicity

For ex vivo cytotoxicity analysis of ICB R, ICB NR, and C Gl261 TILs, a lactate dehydrogenase (LDH) release assay was applied (Promega; G1780). A total of 5 × 10^3^ Gl261 cells were seeded onto 96-well flat bottom plates and incubated at 37 °C, 5% CO_2_ over night to allow for tumor cell adherence. For isolation and purification of TILs from ICB R, ICB NR, and C-treated Gl261 tumors on day 27 post inoculation, tumor-bearing hemispheres were processed to single-cell suspensions, and TILs were purified with myelin removal beads II (Miltenyi Biotec; 130-096) and MagniSort™ Mouse CD3 Positive Selection Kit (eBioscience; 8802-6840) according to the manufacturer’s instruction. Purified TILs were co-cultured with Gl261 cells at a ratio of 10:1 (5 × 10^4^ CD3^+^T − 5 × 10^3^ Gl261 cells) at 37 °C, 5% CO_2_ for 4 h. A total of 4–5 ICB R, ICB NR, and C TIL samples per group were pooled for cytotoxicity analysis. LDH release of TIL-mediated Gl261 killing was assessed with the CytoTox 96® Non-Radioactive Cytotoxicity Kit (Promega) according to the manufacturer’s instruction and OD was measured on a iMarkTM Microplate reader (BioRad; Hercules, Calif.) at 490 nm. Values were corrected for spontaneous effector and target cell LDH release and cell culture medium background. Data are represented as tumor cell lysis relative to positive Gl261 lysis control.

### NanoString analysis and inflammatory gene signatures

RNA from FACS sorted macrophages (CD45^high^CD11b^+^) of Gl261 tumors of ICB R and ICB NR mice was extracted using the PicoPureTM RNA Isolation Kit (Arcturus; KIT0202) and gene expression analysis was performed using the nCounter Mouse Immunology Panel (NanoString; XT-CSO-MIM1-12) with the nCounter NanoString™ technology (NanoString Technologies; Seattle, WA)^57^. RNA input per sample was 25 ng. Data analysis were performed by nSolver 3.0. Marker for pro- and anti-inflammatory gene signatures were selected according to previously described marker. Gene signature scores were calculated as geometric mean of each gene expression. Controls and low count genes were removed from the NanoString count matrix, followed by a scalar normalization and variance modeling^58,59^. Differential gene expression analysis was performed by an eBayes adjusted moderated *t*-statistic linear regression model^60^. Pro- and anti-inflammatory metagene signatures were generated from previously reported markers (Supplementary Table 1) and the geometric mean was estimated in each sample for the different signatures.

### CIBERSORT analysis human glioblastoma

CIBERSORT analysis was applied to expression data of pre-ICB (pembrolizumab/nivolumab) GBM tissue from Zhao et al.^25^. Expression data from GBM samples that were obtained more than 6.5 months prior to the first ICB therapy (pembrolizumab/nivolumab) were excluded. Response classification of patients was adapted from Zhao et al.^25^. In detail, response criteria were met when samples after PD-1 inhibitor therapy showed signs of pseudoprogression (inflammatory response with very few or no tumor cells detectable) or stable or continually shrinking tumor lesions over a minimum of 6 months as detected by MRI^25^. RNAseq *.fastq files for selected patients were downloaded from the ENA using the Aspera Connect client. Reads were aligned to the human genome (GRCh38) using STAR (2.7.0c), and a gene expression matrix (as TPM) was generated using RSEM. The gene expression matrix was analyzed by CIBERSORT using the LM22 signature gene file of 22 immune cell types^61^. Immune cell subtype proportions were compared using Wilcoxon rank-sum test (WRST) and false discovery rate adjustment was performed by Benjamini and Hochberg correction.

### Immunogram ICB response

Fold changes (R/NR) of response features were log2 transformed and *z* transformation for all features was applied. Data are presented as a radar chart.

### Statistics

Data are represented as individual values or as mean ± SEM. Group sizes (*n*) and applied statistical tests are indicated in figure legends. Significance was assessed by either unpaired *t*-test analysis, paired *t*-test analysis,or one-way analysis of variance (ANOVA) analysis with Tukey, Dunnett or Sidak post hoc testing as indicated in figure legends. Spearman correlation was applied for all correlation analysis and the Kaplan–Meier method was used to examine survival differences. Statistics were calculated using GraphPad Prism 7.0.

### Reporting summary

Further information on research design is available in the Nature Research Reporting Summary linked to this article.

## Supplementary information

Supplementary Information

Description of Additional Supplementary Files

Supplementary Data 1

Supplementary Data 2

Reporting Summary

## Data Availability

RNA-seq data that support the findings of this study has been deposited in the GEO repository (GSE129877) and will be made available prior to publication. All additional data sets generated or analyzed during this study are included in this published article and supplementary information files. Data underlying CIBERSORT analysis was published by Zhao J et al.^25^. (Nat. Med., 2019) and was accessed via the GEO repository (GSE121810). The source data underlying Figs. 1b–j, 2a–f, 3a–h, 4a–c, 4e, f, 5a–f, and Supplementary Figs. 1a–g, 2a–c, 3a, 3c, d, 4a, 4c–f, 6a–g, 7a, b, 8a–c, 9a–c, 10a–d, and 11b–f are provided as a Source Data file.

## Code availability

The full codes of all scripts are available on reasonable request. For further information on software package versions please refer to the Nature Research Reporting Summary linked to this article.
