# Supplementary material for: Heterogeneity of response to immune checkpoint blockade in hypermutated experimental gliomas
Source: Nat Commun. 2020 Feb 18;11:931. doi: 10.1038/s41467-020-14642-0 (PMC7028933; doi:10.1038/s41467-020-14642-0)
Supplement: Supplementary file 1 — Supplementary Information [file 41467_2020_14642_MOESM1_ESM.pdf]

Supplementary Information

Heterogeneity of response to immune checkpoint blockade in hypermutated experimental gliomas

Aslan et al.

Supplementary Figures

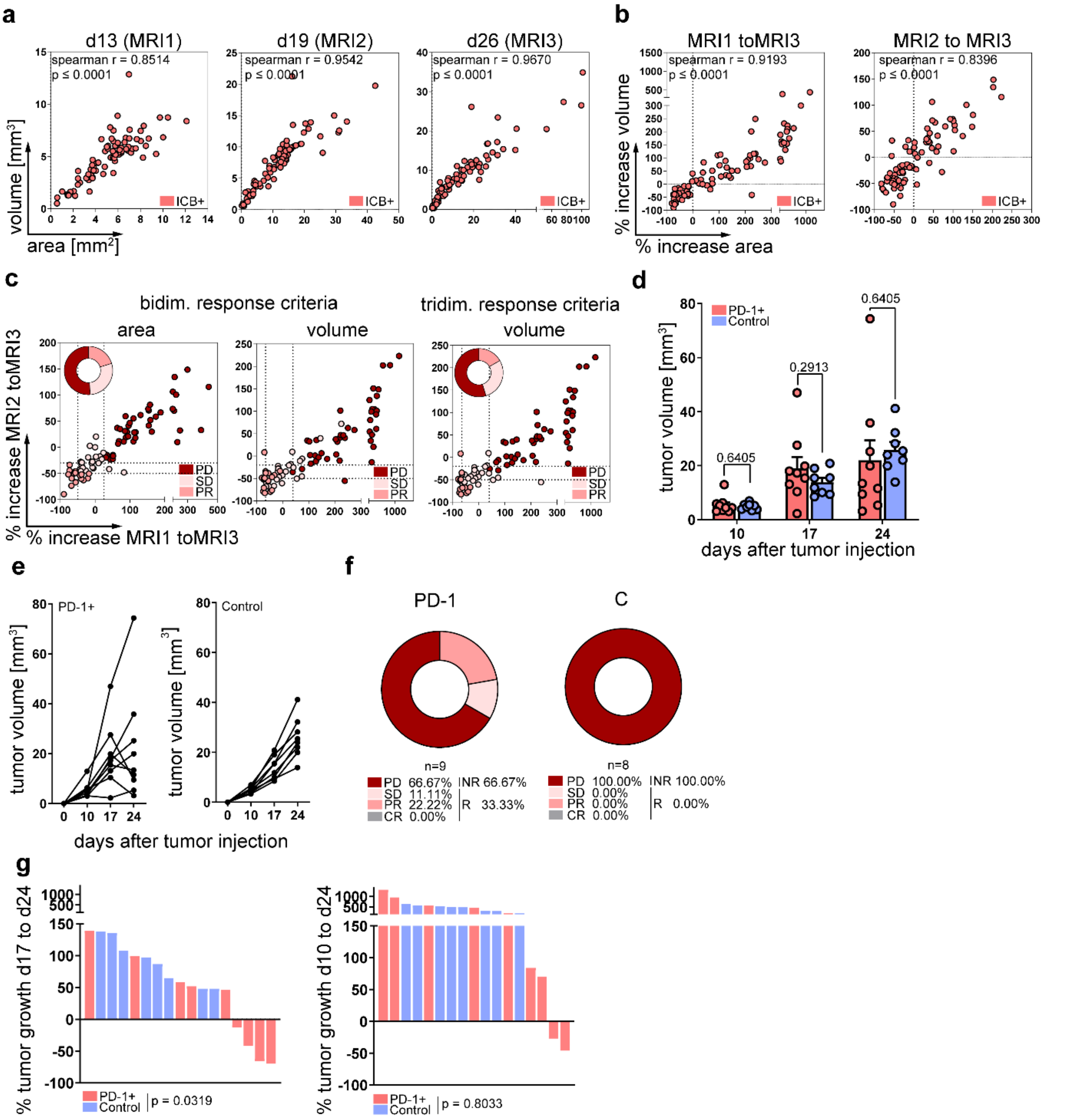

Supplementary Figure 1: Preclinical response criteria for ICB therapy in Gl261 tumors. a-c, C57Bl/6J mice were treated with 250 µg anti-PD-1 and 100 µg anti-CTLA-4 (ICB+) or isotype control (C) every third day for three doses and tumor growth was monitored by MRI before, during and after ICB therapy (MRI1, MRI2 and MRI3, n = 84 animals). **a**, Correlation of bidimensional product of the diameter [mm²] and lesion volume [mm³] on MRI1 (left), MRI2 (middle) and MRI3 (right). **b**, Correlation of % increase in lesion area and lesion volume between MRI1 and MRI3 (left) and MRI2 and MRI3 (right). **c**, Relative increase in lesion area (left) and volumes (middle) between MRI1 and MRI3 and MRI2 and MRI3 of ICB PR,SD and PD tumors defined based on RANO criteria using lesion area (bidimensional). CR: % $A_{MRI3\text{-}MRI1}$ -100 %, PR: % $A_{MRI3\text{-}MRI1} \leq$ -50 %, SD: % $A_{MRI3\text{-}MRI1}$ > -50 % and < +25 %, PD: % $A_{MRI3\text{-}MRI1} \geq$ +25 %. (Right) Relative increase in volumes between MRI1 and MRI3 and MRI2 and MRI3 of ICB PR, SD and PD tumors defined based on RANO criteria using lesion volumes (tridimensional). CR: % $V_{MRI3\text{-}MRI1}$ -100 %, PR: % $V_{MRI3\text{-}MRI1}$ ≤ -65.0 % or % $V_{MRI3\text{-}MRI2}$ ≤ -65.0 %, SD: % $V_{MRI3\text{-}MRI1}$ > -65.0 % and < +40.0 % or % $V_{MRI3\text{-}MRI1} \geq$ +40.0 % and % $V_{MRI3\text{-}MRI2}$ ≤ -30% (unconfirmed progression, UCP), PD: % $V_{MRI3\text{-}MRI1}$ ≥ +40.0 %. **d-g**, C57Bl/6J mice were treated with 250 µg anti-PD-1 or isotype control (C) on d10, d13 and d16 and tumors were monitored by MRI on d10, d17 and d24 post-intracranial Gl261 injection (n = 9 vs. n = 8 animals). Tumor growth (**d**+**e**) and therapy response (**f**+**g**) of anti-PD-1+ and control-treated mice. Response was assessed by the percentage of tumor growth or regression (tridimensional) between d17 and d24 as well as d10 and d24 post-

tumor inoculation. Data are represented as mean ± SEM for **d**. Statistical significance was determined by spearman correlation for **a** and **b.** Statistical significance was determined by two-tailed student's test for **d** and **g**. Source data are provided as a Source Data file.

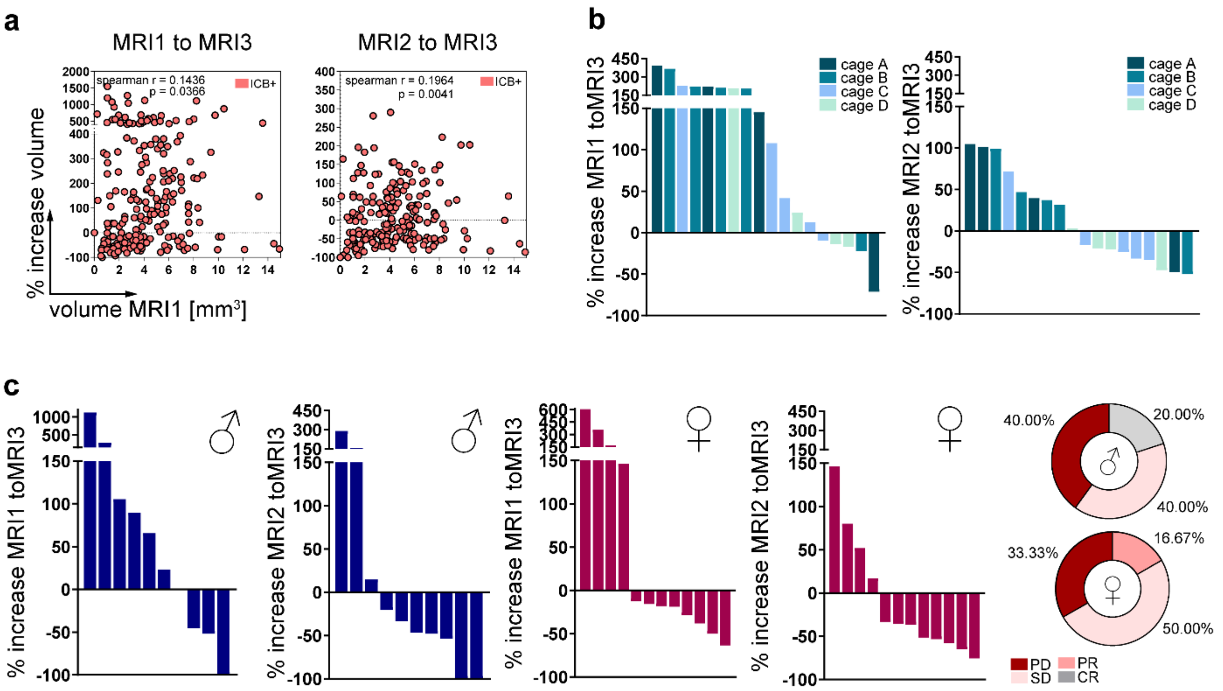

Supplementary Figure 2: ICB response associated factors. a, C57Bl/6J mice were treated with 250 µg anti-PD-1 and 100 µg anti-CTLA-4 (ICB+) or isotype control (C) every third day for three doses and tumor growth was monitored by MR imaging before, during and after ICB therapy (MRI1, MRI2 and MRI3). Correlation of baseline tumor volume and ICB therapy response was determined by spearman correlation of % $V_{MRI3-MRI1}$ (left) and V_{MRI1} and % $V_{MRI3-MRI2}$ and V_{MRI1} (right) (n = 212 animals). **b,** Relative increase in lesion volumes between MRI1 and MRI3 (%$V_{MRI3-MRI1}$) and MRI2 and MRI3 (%$V_{MRI3-MRI2}$) of ICB treated mice in between cages (Cage A n = 4; cage B n = 5, cage C n = 5, cage D n = 4 animals). **c,** Relative increase in lesion volumes between MRI1 and MRI3 (%$V_{MRI3-MRI1}$) and MRI2 and MRI3 (%$V_{MRI3-MRI2}$) of male (♂, left) and female (♀, middle) ICB treated mice (male n = 10, female n = 12 animals). Right: response of ICB treated male and female treated mice. Statistical significance was determined by spearman correlation for **a**. Source data are provided as a Source Data file.

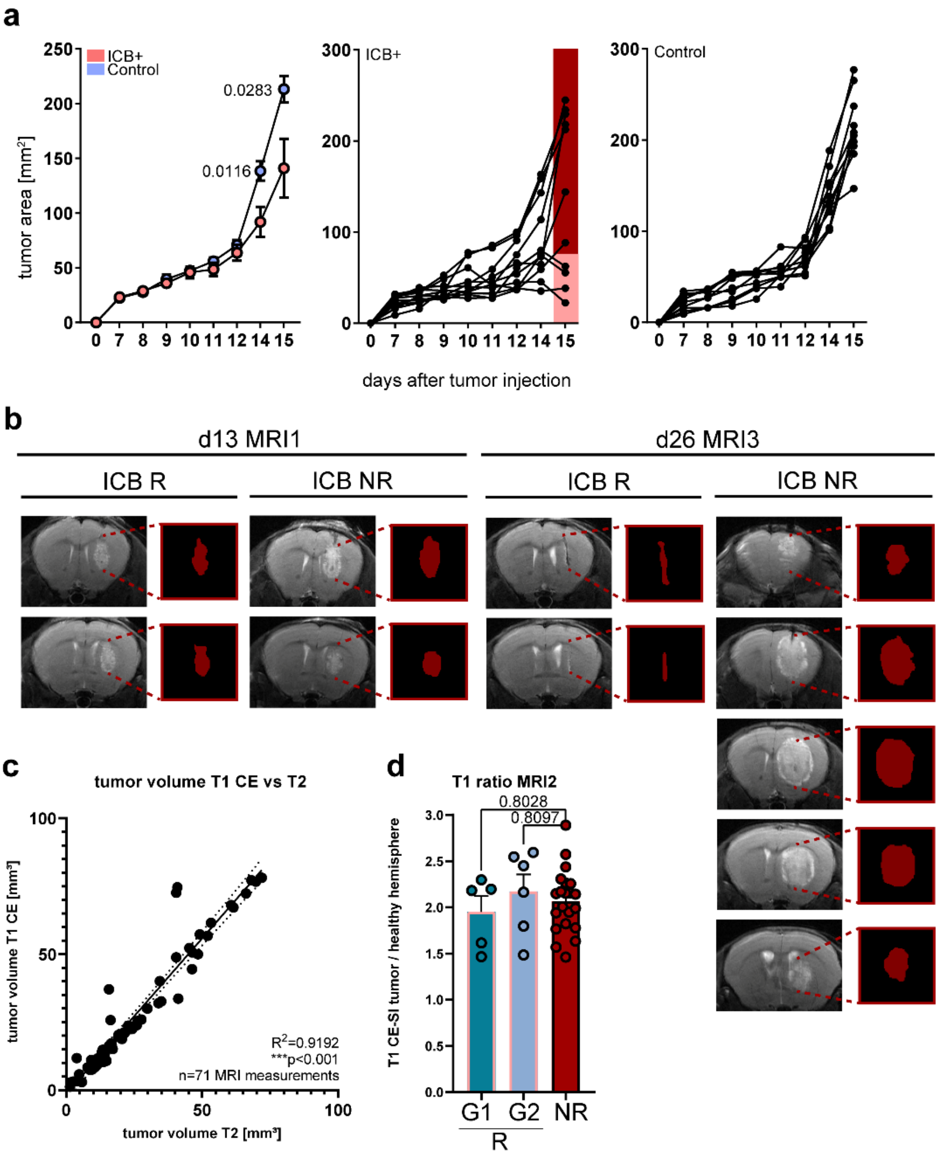

Supplementary Figure 3: Radiomics analysis of ICB R and ICB NR. a, C57BL/6J mice were treated with 250 µg anti-PD-1 and 100 µg anti-CTLA-4 (ICB+) or isotype control (C) at day 7, 10 and 13 after subcutaneous B16 tumor injection (ICB n = 12 vs. C n = 10 animals) and tumor diameters were measured daily/every second day. ICB+ tumors were grouped into regressing/stable tumors (light red) and progressing tumors (dark red). **b**, Representative images of segmentation of MRI data for radiomic signature analysis. **c**, correlation of T1 CE and T2 Gl261 tumor volumes of ICB R, ICB NR and C mice (n=71 MRI measurements). **d**, Ratio T1 CE-SI tumor / healthy hemisphere of directly responding ICB R (G1), pseudoprogressing ICB R (G2) and ICB NR at MRI2. (ICB R G1 n=5, ICB R G2 n=6, ICB NR n=21 animals). Data are represented as mean ± SEM for **a** and **d**. Statistical significance was determined by two-tailed student's t test for **a,** pearson correlation for **c** or by one-way ANOVA in combination with Tukey's test for **d**. Source data are provided as a Source Data file.

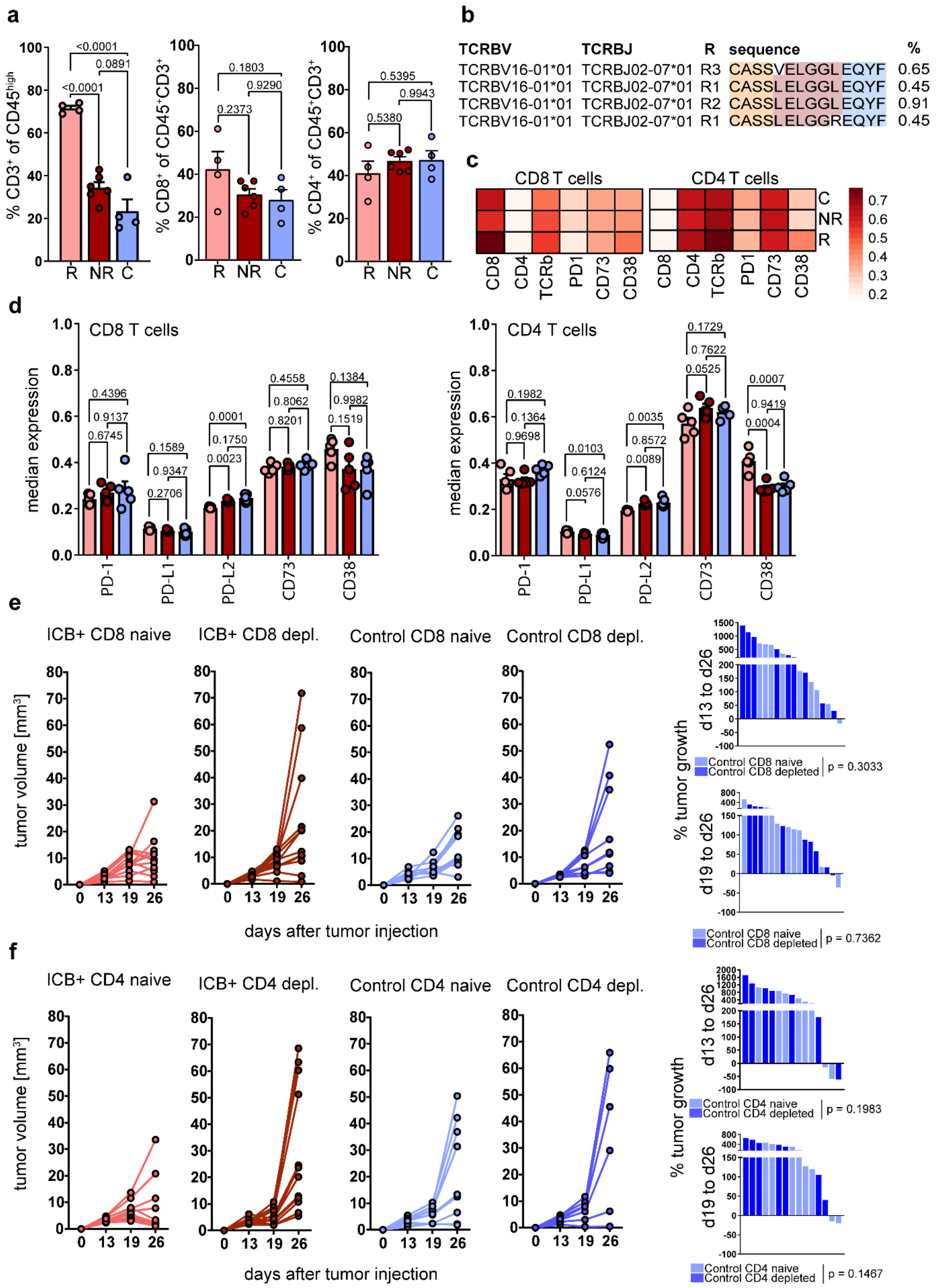

Supplementary Figure 4: anti-tumor T cell immunity in ICB R and ICB NR tumors. C57BL/6J mice were treated with 250 µg anti-PD-1 and 100 µg anti-CTLA-4 (ICB+) or isotype control (C) on d13, d16 and d19 after intracranial Gl261 tumor injection. **a**, Flow cytometry analysis for frequency of CD3^{+} TILs of CD45 cells (left) and CD8^{+} T cells (middle) and CD4^{+} T cells (right) of CD3^{+} TILs from ICB R, ICB NR and C tumors (ICB R n = 3, ICB NR n = 6, C n = 4 animals). **b**, CD8 TCRβ sequence motif (alteration 1 or less aa) exclusively identified in ICB R CD8 TIL subset by TCRβ GLIPH analysis (ICB R n=3; ICB NR n=3 animals). **c-d,** Median

expression of CD8, CD4, TCRβ, PD-1, CD73 and CD38 on CD8$^+$ and CD4$^+$ TILs from ICB R, ICB NR and C mice assessed by multi-parameter flow cytometry (ICB R n = 5, ICB NR n = 5, C n = 5 animals). **e-f**, CD8$^+$ or CD4$^+$ T cells were depleted prior to and during ICB therapy using monoclonal depletion antibodies (4 x 500 µg 2.43 2 x 1000 µg GK1.5). **e**, Tumor growth and response to ICB therapy in CD8 depleted and naïve mice (ICB+ CD8 naïve n = 13, ICB+ CD8 depl. n = 13, C CD8 naïve n = 9, C CD8 depl. n = 9 animals). **f**, Tumor growth and response to ICB therapy in CD4 depleted and naïve mice (ICB+ CD4 naïve n = 12, ICB+ CD4 depl. n = 12, C CD4 naïve n = 8, C CD4 depl. n = 7 animals). Data are represented as mean ± SEM for **a**+**d**. Statistical significance was examined by one-way ANOVA in combination with Tukey's test for **a**+**d** or by two-tailed student's t test for **e** and **f**. Source data are provided as a Source Data file.

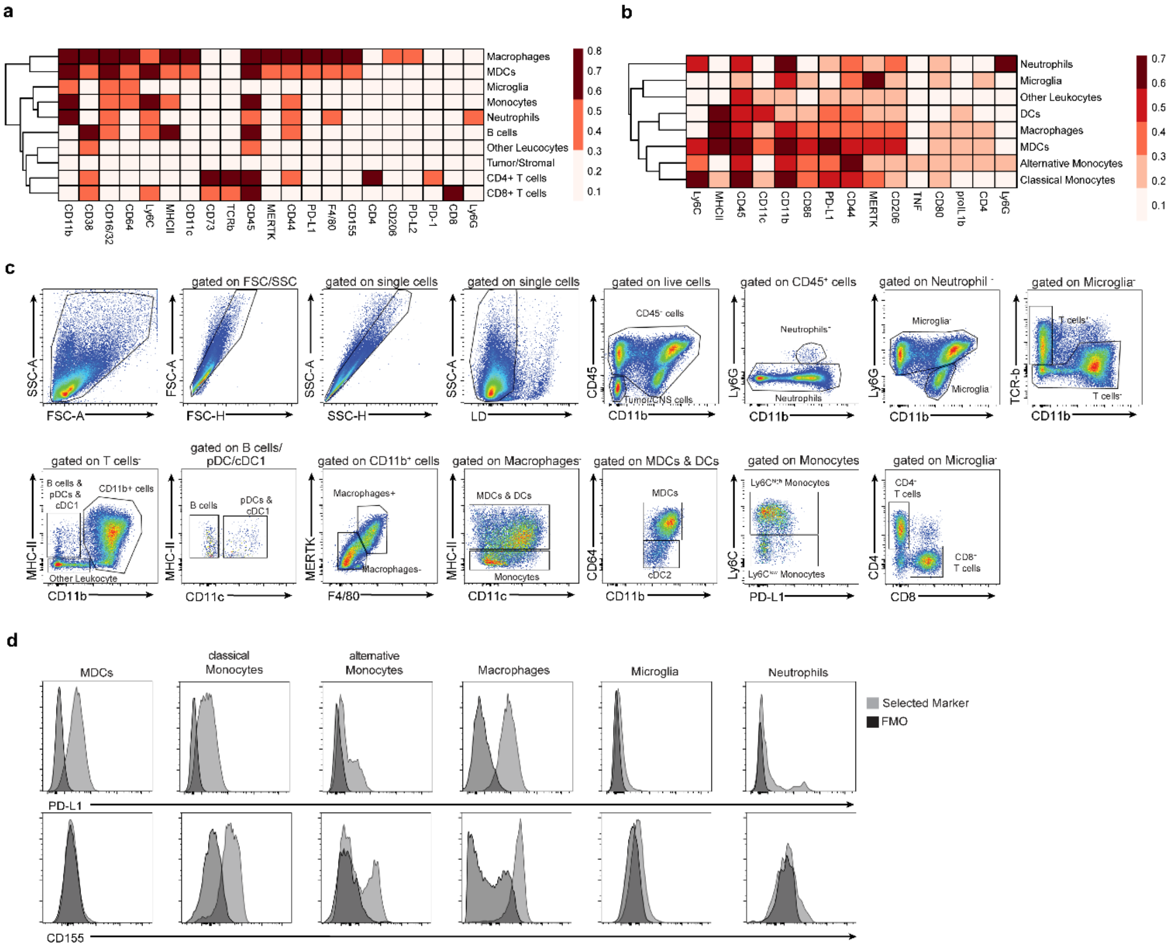

Supplementary Figure 5: tSNE-guided immune cell subtyping in ICB R and ICB NR CNS. a-d, C57Bl/6J mice were treated with 250 µg anti-PD-1 and 100 µg anti-CTLA-4 (ICB+) or isotype control (C) on d13, d16 and d19 and tumor growth was monitored by MR imaging on d13, d19 and d26 post intracranial Gl261 tumor injection (ICB R n = 5, ICB NR n = 5, C n = 5 animals) **a**, Cell subtyping of CNS infiltrating cells from ICB R, ICB NR and C mice. Heatmap showing the median expression of indicated markers (value range: 0-1, white - red) of the FlowSOM-guided meta-clustering on living and single cells. **b**, Myeloid cell subtyping of CNS infiltrating cells from ICB R, ICB NR and C mice. Heatmap of the median expression of indicated markers (value range: 0-1, white - red) of the FlowSOM-guided meta-clustering on living and single $CD45^{+}CD11b^{+}$ myeloid cells. **c**, Manual gating of flow cytometry data on major leukocyte populations present in a representative brain sample among Gl261-bearing mice treated with isotype control antibodies. **d**, Manual gating of expression levels of PD-L1 and CD155 in MDCs, classical and alternative monocytes, macrophages, microglia and neutrophils (light grey) compared to respective FMO controls (dark grey).

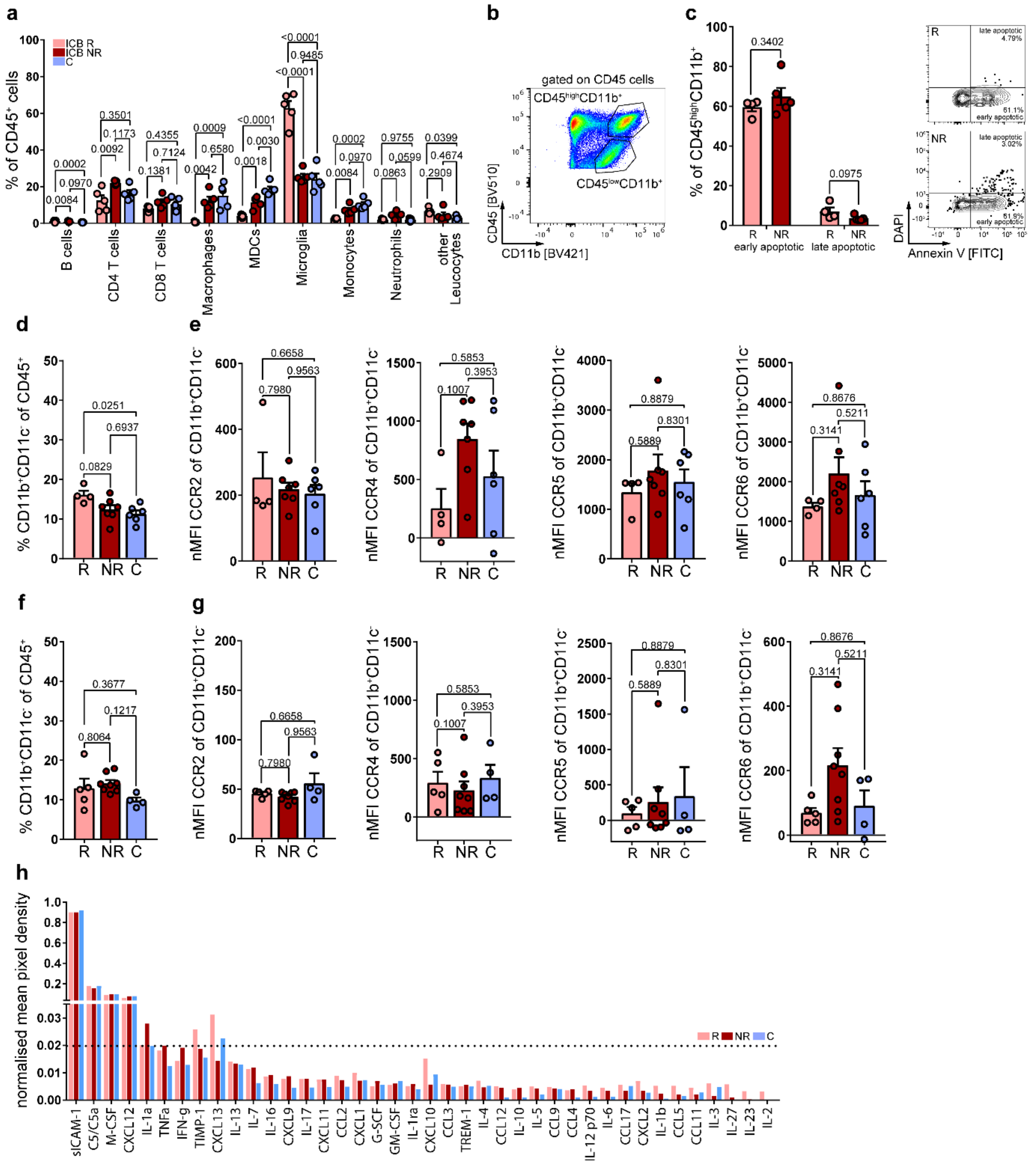

Supplementary Figure 6: Migration of circulating CD11b+ cells and apoptosis of tumor-associated CD45^{high}CD11b+ cells during ICB therapy. **a-f**, C57Bl/6J mice were treated with 250 µg anti-PD-1 and 100 µg anti-CTLA-4 (ICB+) or isotype control (C) on d13, d16 and d19 and tumor growth was monitored by MR imaging on d13, d19 and d26 post intracranial Gl261 tumor injection. **a**, tSNE-guided cell subtyping (relative frequencies) of CNS infiltrating cells from ICB R, ICB NR and C mice (ICB R n = 5, ICB NR n = 5, C n = 5 animals). **b**, representative gating of CD45^{high}CD11b^{+} cells in Gl261 tumors. Cells were pre-gated for singlets, viable cells and CD45^{+} cells. **c**, apoptosis analysis of tumor-associated CD45^{high}CD11b^{+} cells by flow cytometry (ICB R n = 4, ICB NR n = 5 animals). **d-g,** Flow cytometry analysis from blood of ICB responder (R), non-responder (NR) and control-treated (C) mice was performed of circulating CD11b^{+}CD11c^{-} cells on d15 (**d**) and d21 (**f**) after tumor injection. Normalized mean fluorescence intensity (nMFI) of CCR2, CCR4, CCR5 and CCR6 on CD11b^{+}CD11c^{-} cells on d15 (**e**) and d21 (**g**) after tumor injection. (d15: ICB R n = 4, ICB NR n = 7, C n = 6; d21: ICB R n = 5, ICB NR n = 8, C n = 4 animals) **h**, Plasma levels of cytokines and chemokines of ICB responder (R), non-responder (NR) and control-treated (C) pooled blood samples at day 15 after tumor injection.). Data are represented as mean ± SEM for **a** and **c**-**g**. Statistical significance was examined by one-way ANOVA in combination with Tukey's test for **a**, **d-g** or by two-tailed student's t test for **c**. Source data are provided as a Source Data file.

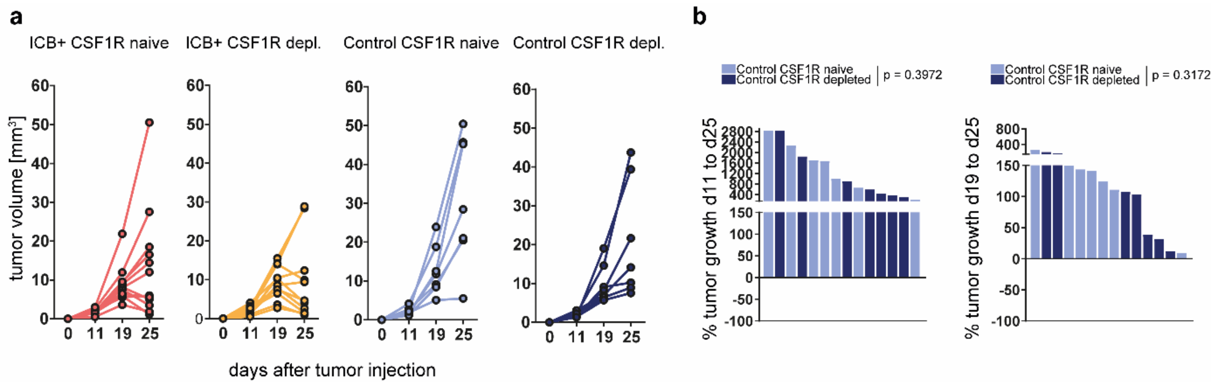

Supplementary Figure 7: combination of CSF1R depletion and ICB therapy in Gl261 tumors. **a-b**, CSF1R was targeted prior and during ICB therapy using monoclonal antibodies (AFS98; 6 x 250 µg). Tumor growth (**a**) and response (**b**) to ICB therapy in CSF1R-targeted and control mice (ICB+ CSF1R naive n = 12, ICB+ CSF1R depleted n = 11; C CSF1R naïve n = 7, C CSF1R depleted n = 7 animals). All data are represented as individual data points. Statistical significance was examined by two-tailed student's t test for **b**. Source data are provided as a Source Data file.

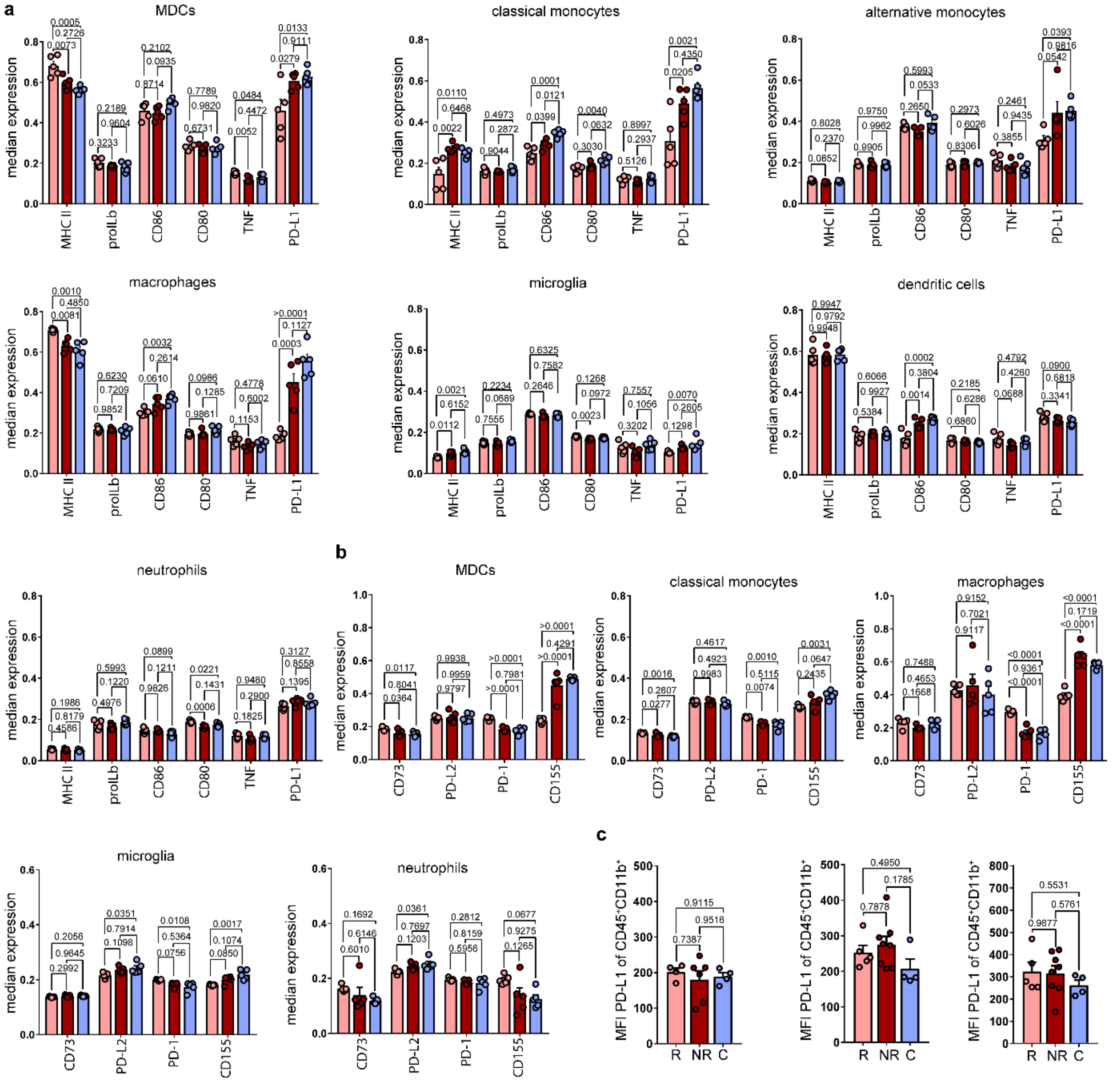

Supplementary Figure 8: tSNE-guided myeloid cell phenotyping in ICB R and ICB NR tumors. a-c, C57Bl/6J mice were treated with 250 µg anti-PD-1 and 100 µg anti-CTLA-4 (ICB+) or isotype control (C) on d13, d16 and d19 and tumor growth was monitored by MR imaging on d13, d19 and d26 post intracranial Gl261 tumor injection. **a**, Median expression of activation marker and PD-L1 on MDCs, classical and alternative monocytes, macrophages, microglia, neutrophils and dendritic cells of ICB R, ICB NR and C tumors assessed by multi-parameter flow cytometry (ICB R n = 5, ICB NR n = 5, C n = 5 animals). **b**, Median expression of suppressive surface molecules on MDCs, classical and alternative monocytes, macrophages and microglia cell cluster of ICB R, ICB NR and C tumors assessed by multi-parameter flow cytometry (ICB R n = 5, ICB NR n = 5, C n = 5 animals). **c**, PD-L1 expression assessed by flow cytometry on ICB R, ICB NR and C on splenic $CD11b^+$myeloid cells (left) and blood $CD11b^+$myeloid cells (d15 middle; d21 right) (spleen: ICB R n = 4, ICB NR n = 6, C n = 4; blood: ICB R n = 5, ICB NR n = 8, C n = 4 animals). All data are represented as mean ± SEM. Statistical significance was determined by one-way ANOVA in combination with Tukey's test for **a-c.** Source data are provided as a Source Data file.

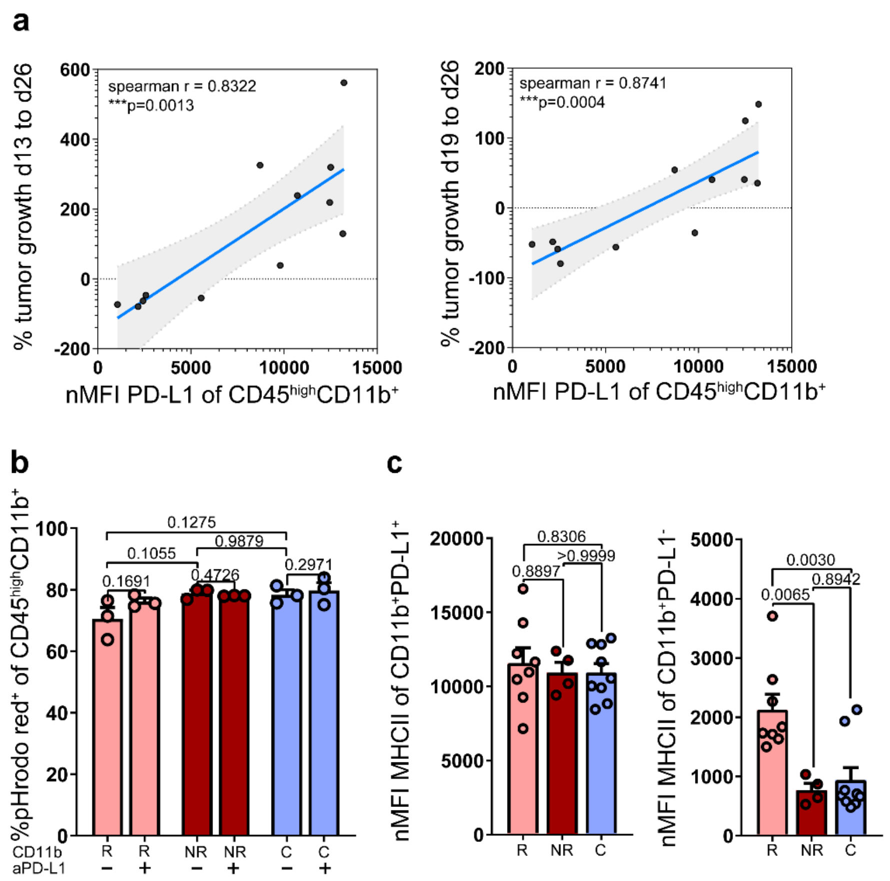

Supplementary Figure 9: Immunosuppressive phenotype of tumor-associated myeloid cells in ICB NR mice. a-c, C57Bl/6J mice were treated with 250 µg anti-PD-1 and 100 µg anti-CTLA-4 (ICB+) or isotype control (C) on d13, d16 and d19 and tumor growth was monitored by MR imaging on d13, d19 and d26 post intracranial Gl261 tumor injection. **a**, correlation of ICB response with PD-L1 expression on tumor-associated $CD45^{high}CD11b^{+}$ cells (n = 12 animals). **b**, *Ex vivo* phagocytosis of tumor-associated $CD45^{high}CD11b^{+}$ cells from ICB R, ICB NR and C mice with and without 20 µg ml^{-1} anti-PD-L1 (10F.9G2) assessed by flow cytometry for pHrhodo red^{+} cells (ICB R n = 3, ICB NR n = 3, C n = 3 animals). **c**, mean MHC II expression on $PD\text{-}L1^{+}$ and $PD\text{-}L1^{-}$ $CD11b^{+}$ cells from ICB R, ICB NR and C tumors assessed by flow cytometry (ICB R n = 8, ICB NR n = 4, C n = 9 animals). Data are represented as mean ± SEM for **b**-**c**. Statistical significance was assessed by spearman correlation for **a**, by one-way ANOVA in combination with Tukey's test for **b** and **c** and paired two-tailed student's t test for **b** (-PD-L1 vs. +PD-L1). Source data are provided as a Source Data file.

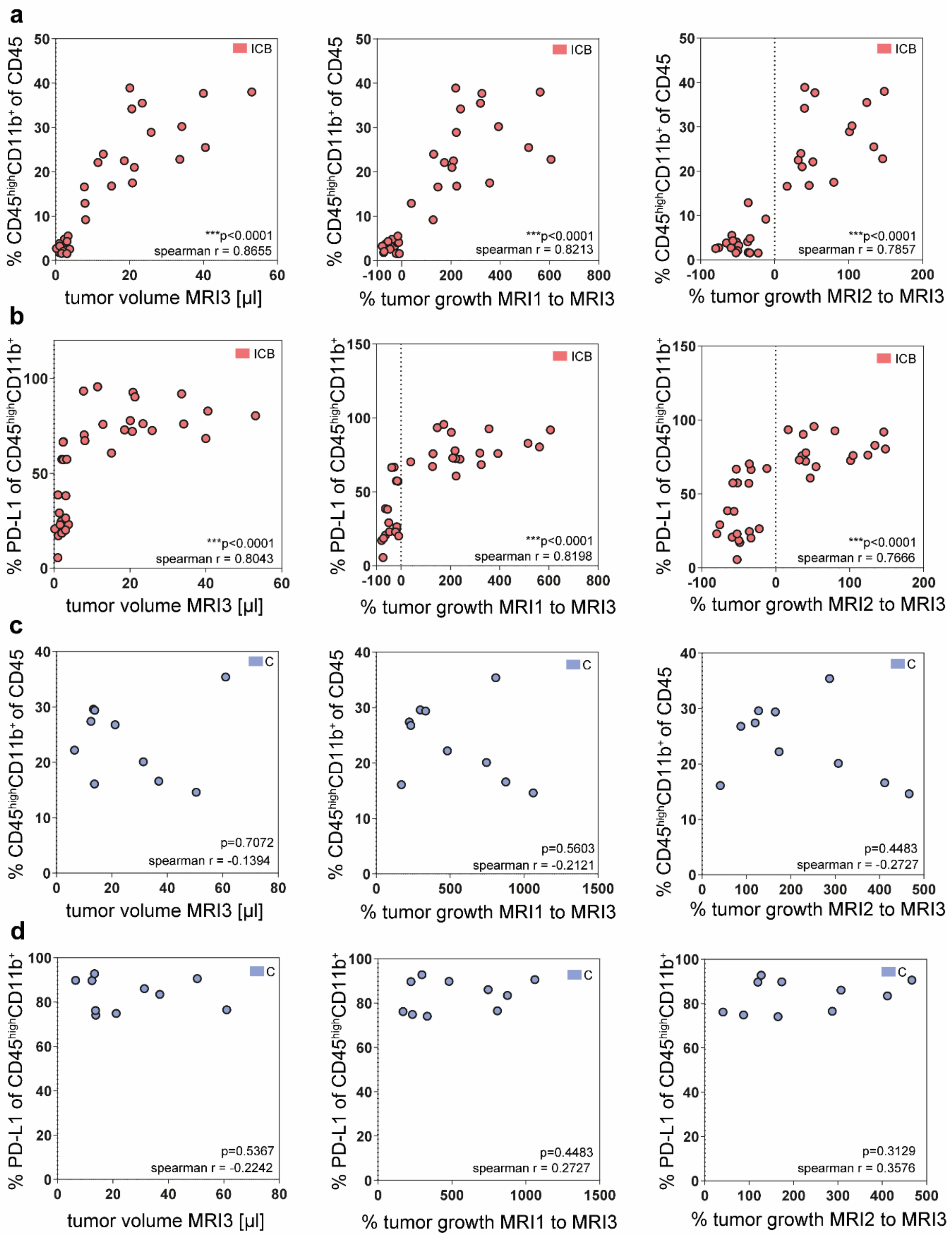

Supplementary Figure 10: Immunosuppressive phenotype of tumor-associated myeloid cells is associated to ICB resistance. a-d, C57Bl/6J mice were treated with 250 µg anti-PD-1 and 100 µg anti-CTLA-4 (ICB+) or isotype control (C) on d13, d16 and d19 and tumor growth was monitored by MR imaging on MRI, MRI2 and MRI3 post intracranial Gl261 tumor injection. **a-b**, correlation of MRI3 tumor volumes (left), tumor growth MRI1 to MRI3 (middle) and tumor growth MRI2 to MRI3 (right) from ICB mice with percentage of tumor-associated $CD45^{high}CD11b^{+}$ cells (**a**) or PD-L1 expression on tumor-associated $CD45^{high}CD11b^{+}$ cells (**b**). **c-d**, correlation of MRI3 tumor volumes (left), tumor growth MRI1 to MRI3 (middle) and tumor growth MRI2 to MRI3 (right) from C mice with percentage of tumor-associated $CD45^{high}CD11b^{+}$ cells (**c**) or PD-L1 expression on tumor-associated $CD45^{high}CD11b^{+}$ cells (**d**) For correlation analysis data from 3 (**a**+**b**, n=35) and 2 (**c**+**d**, n=10) independent experiments were pooled. Statistical significance was assessed by spearman correlation for **a-d**. Source data are provided as a Source Data file.

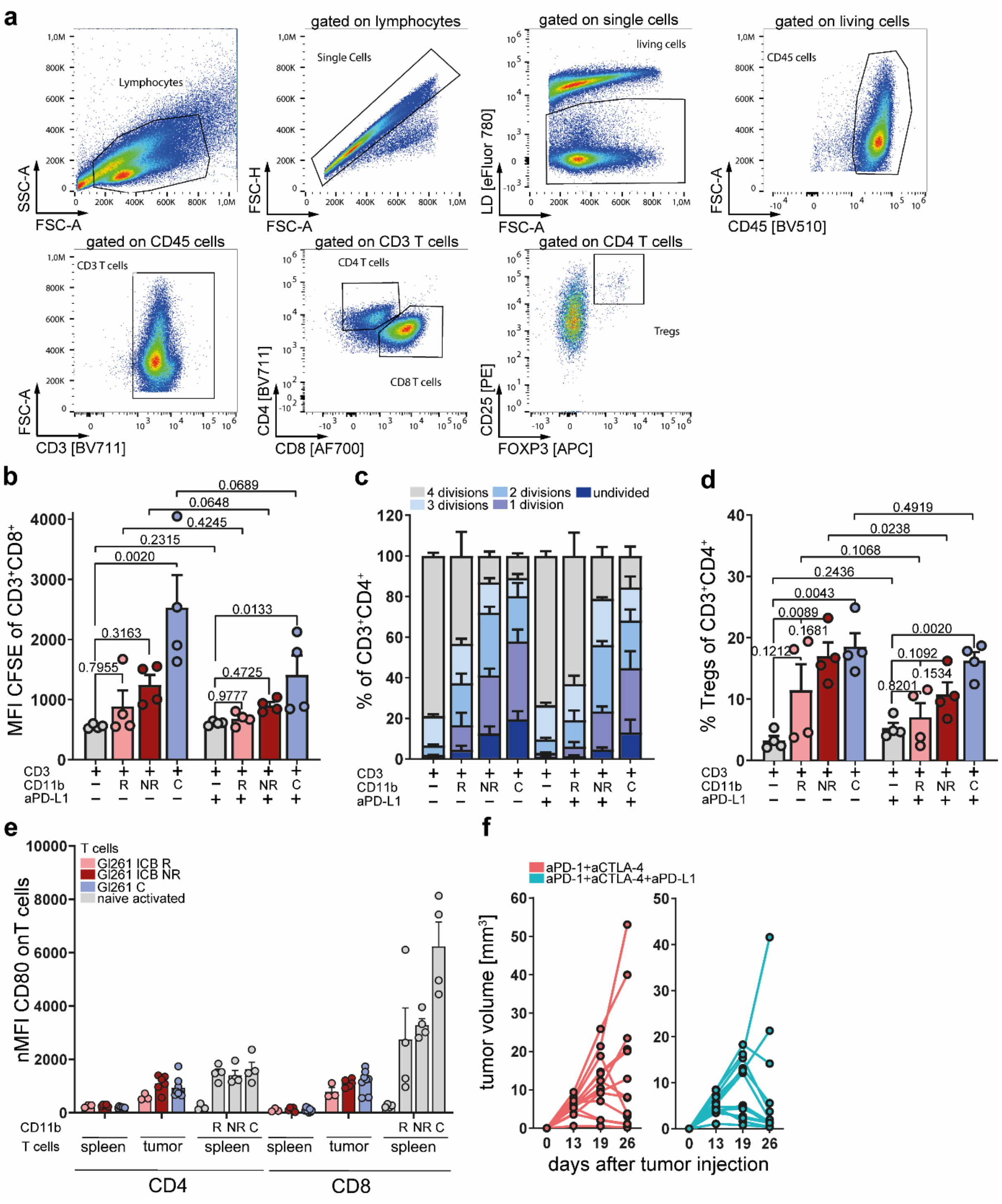

Supplementary Figure 11: Resistance to ICB therapy is mediated by PD-L1 expression on tumor-associated myeloid cells in ICB NR mice. a-d, C57Bl/6J mice were treated with 250 µg anti-PD-1 and 100 µg anti-CTLA-4 (ICB+) or isotype control (C) on d13, d16 and d19 and MR imaging was performed on d13, d19 and d26 post Gl261 injection. **a-d**, *Ex vivo* T cell suppression of tumor-associated myeloid cells from ICB R, ICB NR and C mice. $CD11b^+$ cells were purified from ICB R, ICB NR and C tumors on d27 post-tumor inoculation by MACS and co-cultured for 72 h with pre-activated naïve $CD3^+$ splenocytes with and without 20 µg ml^{-1} anti-PD-L1 (10F.9G2) (ICB R n = 4, ICB NR n = 4, C n = 4 animals). **a**, gating strategy for T cell suppression assay. **b-d**, $CD8^+$ (**b**) and $CD4^+$ (**c**) T cell proliferation and $CD25^+FOXP3^+$ T_{reg} frequencies (**d**) after 72 h co-culture with tumor-associated myeloid cells from ICB R, ICB NR and C mice assessed by CFSE staining. **e**, CD80 expression was analyzed on splenocytes and TILs from ICB R, ICB NR and C tumors and pre-activated splenocytes from naïve mice co-cultured with ICB R, ICB NR and C tumor-associated CD11b+ cells by flow cytometry (spleen and TILs: ICB R n = 3, ICB NR n = 5, C n = 8; spleen naïve: T cells only n = 4, T cells + ICB R CD11b n=4, T cells + ICB NR CD11b n = 4, T cells + C CD11b n = 4). **f**, C57BL/6J mice were treated

with 250 µg anti-PD-1 and 100 µg anti-CTLA-4 and as combinatory therapy with additional 200 µg anti-PD-L1 on d13, d16 and d19 post-tumor inoculation and tumor growth was assessed by MRI. (aPD-1 + aCTLA-4 n = 13, aPD-1 + aCTLA-4 + aPD-L1 n = 13 animals). Data are represented as mean ± SEM. For **b** statistical significance was determined by one-way ANOVA in combination with Dunnett's test ($CD3^+$ cells + R, NR or C $CD11b^+$ cells vs. T cells only) or two-tailed paired student's test (-PD-L1 vs. + PD-L1). For **d** statistical significance was analyzed by one-way ANOVA in combination with Dunnett's test ($CD3^+$ cells + R, NR or C $CD11b^+$ cells vs. T cells only) and Sidak's test ($CD3^+$ cells + R $CD11b^+$ cells vs. $CD3^+$ cells + NR $CD11b^+$ cells) for multiple comparison or two-tailed paired student's t test (-PD-L1 vs. + PD-L1). Source data are provided as a Source Data file.

Supplementary Tables

Supplementary Table 1: pro- and anti-inflammatory gene expression in intratumoral ICB R and NR macrophages.

gene	Signature	NR	NR	NR	R	R	R	R	logFC	adj.p.	Ref
Cd274	anti-inflammatory	7,874	7,884	6,906	9,238	9,569	9,646	9,560	1,932	0,0044	[1,2]
Il1rn	anti-inflammatory	12,315	11,248	12,200	9,959	11,349	9,395	10,075	-1,745	0,0277	[3,4]
Il10rb	anti-inflammatory	10,942	11,159	11,298	10,812	10,979	10,581	10,653	-0,384	0,1010	[5,6]
Il4ra	anti-inflammatory	11,489	11,534	11,996	10,633	10,809	10,523	10,609	-1,034	0,0051	[7]
Il6st	anti-inflammatory	7,379	7,701	6,706	8,210	7,984	8,626	7,951	0,917	0,0489	[8]
Msr1	anti-inflammatory	10,780	9,866	10,686	9,048	9,618	8,653	9,412	-1,230	0,0162	[5,9]
Tgfb1	anti-inflammatory	11,812	11,340	11,366	11,437	11,750	11,059	10,918	-0,232	0,4662	[10,11]
Tgfbi	anti-inflammatory	14,793	14,117	14,673	13,163	13,884	12,874	13,207	-1,240	0,0121	[4,12]
Cd80	pro-inflammatory	8,768	8,378	8,744	8,340	8,205	8,101	8,530	-0,332	0,1875	[13]
Cd86	pro-inflammatory	10,696	10,505	10,707	10,095	10,382	10,164	10,409	-0,371	0,0863	[5,13]
Cxcl10	pro-inflammatory	11,188	10,573	11,191	9,435	10,182	9,619	11,073	-0,893	0,1214	[8,13]
H2-Ab1	pro-inflammatory	15,754	16,224	16,274	15,852	15,498	15,652	15,867	-0,365	0,1315	[3,13]
H2-DMb2	pro-inflammatory	9,157	9,091	8,750	9,904	9,823	9,962	9,412	0,778	0,0156	[3,13]
Il1b	pro-inflammatory	12,937	11,958	13,439	11,159	12,767	10,629	11,975	-1,163	0,1173	[3,5,7]
Il12a	pro-inflammatory	6,570	6,731	6,387	6,836	7,688	7,934	7,634	0,950	0,0311	[3,7,13]
Il6	pro-inflammatory	6,957	5,921	6,162	6,713	7,613	8,020	7,226	1,033	0,0587	[3,7,13]
Nos2	pro-inflammatory	7,350	8,537	8,442	8,768	8,977	9,313	9,425	0,979	0,0587	[3,7,13]
Tlr2	pro-inflammatory	10,898	10,975	11,120	10,358	10,469	10,455	10,789	-0,477	0,0489	[5,8,13]
Tnf	pro-inflammatory	9,169	9,185	8,812	8,814	8,836	9,346	9,127	-0,058	0,8228	[3,5,7]

Gene expression in log2 counts per million and differential gene expression in R vs. NR macrophages. P values were adjusted using Benjamini Hochberg correction.

Supplementary References

1. Hartley, G. P., Chow, L., Ammons, D. T., Wheat, W. H. & Dow, S. W. Programmed Cell Death Ligand 1 (PD-L1) Signaling Regulates Macrophage Proliferation and Activation. *Cancer Immunol Res* **6**, 1260–1273 (2018).
2. Xiong, H. *et al.* Anti-PD-L1 treatment results in functional remodeling of the macrophage compartment. *Cancer Res.* (2019) doi:10.1158/0008-5472.CAN-18-3208.
3. Hu, X. *et al.* Microglial and macrophage polarization—new prospects for brain repair. *Nature Reviews Neurology* **11**, 56 (2015).
4. Darmanis, S. *et al.* Single-Cell RNA-Seq Analysis of Infiltrating Neoplastic Cells at the Migrating Front of Human Glioblastoma. *Cell Rep* **21**, 1399–1410 (2017).
5. Müller, S. *et al.* Single-cell profiling of human gliomas reveals macrophage ontogeny as a basis for regional differences in macrophage activation in the tumor microenvironment. *Genome Biol* **18**, (2017).
6. Shouval, D. S. *et al.* Interleukin-10 receptor signaling in innate immune cells regulates mucosal immune tolerance and anti-inflammatory macrophage function. *Immunity* **40**, 706–719 (2014).
7. Murray, P. J. Macrophage Polarization. *Annual Review of Physiology* **79**, 541–566 (2017).
8. Jablonski, K. A. *et al.* Novel Markers to Delineate Murine M1 and M2 Macrophages. *PLOS ONE* **10**, e0145342 (2015).
9. Kubota, K. *et al.* CD163 + CD204 + tumor-associated macrophages contribute to T cell regulation via interleukin-10 and PD-L1 production in oral squamous cell carcinoma. *Scientific Reports* **7**, 1755 (2017).

10. Wurdinger, T., Deumelandt, K., van der Vliet, H. J., Wesseling, P. & de Gruijl, T. D. Mechanisms of intimate and long-distance cross-talk between glioma and myeloid cells: How to break a vicious cycle. *Biochimica et Biophysica Acta (BBA) - Reviews on Cancer* **1846**, 560–575 (2014).
11. Ye, X. *et al.* Tumor-Associated Microglia/Macrophages Enhance the Invasion of Glioma Stem-like Cells via TGF-β1 Signaling Pathway. *The Journal of Immunology* **189**, 444–453 (2012).
12. Biswas, S. K. *et al.* A distinct and unique transcriptional program expressed by tumor-associated macrophages (defective NF-kappaB and enhanced IRF-3/STAT1 activation). *Blood* **107**, 2112–2122 (2006).
13. Mantovani, A., Sozzani, S., Locati, M., Allavena, P. & Sica, A. Macrophage polarization: tumor-associated macrophages as a paradigm for polarized M2 mononuclear phagocytes. *Trends Immunol.* **23**, 549–555 (2002).
