# Supplementary material for: Heterogeneity of response to immune checkpoint blockade in hypermutated experimental gliomas
Source: Nat Commun. 2020 Feb 18;11:931. doi: 10.1038/s41467-020-14642-0 (PMC7028933; doi:10.1038/s41467-020-14642-0)
Supplement: Supplementary file 2 — Description of Additional Supplementary Files [file 41467_2020_14642_MOESM2_ESM.docx]

**Description of Additional Supplementary Files**

**Supplementary Data 1:** Radiomic features for MRI-based ICB response prediction. List of 423 radiomic Features for non-invasive MRI-based ICB response prediction in Gl261 tumors from Figure 2d-f.

**Supplementary Data 2:** Flow cytometry and histology antibodies. Reactivity, clone, label, LOT and dilution of flow cytometry and immunohistochemistry antibodies
