# Supplementary material for: Heterogeneity of response to immune checkpoint blockade in hypermutated experimental gliomas
Source: Nat Commun. 2020 Feb 18;11:931. doi: 10.1038/s41467-020-14642-0 (PMC7028933; doi:10.1038/s41467-020-14642-0)
Supplement: Supplementary file 5 — Reporting Summary [file 41467_2020_14642_MOESM5_ESM.pdf]

nature research

Corresponding author(s): Michael Platten

Last updated by author(s): Jan 12, 2020

Reporting Summary

Nature Research wishes to improve the reproducibility of the work that we publish. This form provides structure for consistency and transparency in reporting. For further information on Nature Research policies, see Authors & Referees and the Editorial Policy Checklist .

Statistics

For all statistical analyses, confirm that the following items are present in the figure legend, table legend, main text, or Methods section.

n/a	Confirmed	
☐	☒	The exact sample size (*n*) for each experimental group/condition, given as a discrete number and unit of measurement
☐	☒	A statement on whether measurements were taken from distinct samples or whether the same sample was measured repeatedly
☐	☒	The statistical test(s) used AND whether they are one- or two-sided<br>*Only common tests should be described solely by name; describe more complex techniques in the Methods section.*
☒	☐	A description of all covariates tested
☐	☒	A description of any assumptions or corrections, such as tests of normality and adjustment for multiple comparisons
☐	☒	A full description of the statistical parameters including central tendency (e.g. means) or other basic estimates (e.g. regression coefficient) AND variation (e.g. standard deviation) or associated estimates of uncertainty (e.g. confidence intervals)
☒	☐	For null hypothesis testing, the test statistic (e.g. *F*, *t*, *r*) with confidence intervals, effect sizes, degrees of freedom and *P* value noted<br>*Give P values as exact values whenever suitable.*
☒	☐	For Bayesian analysis, information on the choice of priors and Markov chain Monte Carlo settings
☒	☐	For hierarchical and complex designs, identification of the appropriate level for tests and full reporting of outcomes
☒	☐	Estimates of effect sizes (e.g. Cohen's *d*, Pearson's *r*), indicating how they were calculated

Our web collection on statistics for biologists contains articles on many of the points above.

Software and code

Policy information about availability of computer code

Data collection	Flow cytometry data was acquired using the BD FACSDIVA software (version 9) or Attune NxT Software (version 2.5). Microscopy data was collected using the ZEISS ZEN software. MRI data was analysed with Osrix, ImageJ and Amira imaging software.
Data analysis	Flow cytometry data was analyzed using FlowJo V9 or V10. For multi-parameter flow cytometry analysis, data was compensated, exported (FlowJo V10), uploaded and normalized using Cyt3 (Matlab_R2018b). The new generated FCS files were uploaded in Rstudio (Version 1.1.463). tSNE (displaying stochastically selected events from all different conditions) and FlowSOM (events from each condition) were performed as described by Brumelman et al. (Nat. Protocol in press).Quantification of microscopy images was done using ImageJ. Tumor volumes from MRI T2w and T1w imaging after Gd-contrast injection were calculated after manual segmentation of tumor areas using Osirix, ImageJ or Amira imaging software. Radiomic features were calculated using medical imaging interaction toolkit (MITK). Analysis of TCR repertoires were performed with the immunoSEQ Analyzer (Adaptive Biotechnlologies, version 3.0). NanoString RNA seq data was analyzed using R (version 3.4.4, 2018-03-15), Bioconductor 3.6 (BiocInstaller 1.28.0), AnnotationDbi_1.40.0, biomaRt_2.34.2, edgeR_3.20.9, ggplot2_3.1.0, ggpubr_0.2, limma_3.34.9 and RColorBrewer_1.1-2. Platform:x86_64-pc-linux-gnu (64-bit), running under: Ubuntu 16.04.5 LTS.<br>CIBERSORT was performed using Stanford web server. For tumor exome sequencing, vendor specific sequencing data format generated by the Illumina NextSeq500 was converted to a standard file format by the Illumina tool bcl2fastq(v2.15.0.4). Sequencing read quality analysis was performed with fastqc (v0.10.1) and alignment was performed with bwa mem (v0.7.5) and the mouse reference genome GRCm38.68. Picard-tools (v1.105) was used to remove duplicates from the alignment files. Sorting and indexing of these files was done with samtools (v0.1.19). Variants were called by samtools mpileup (v0.1.19) for single nucleotide variants and platypus (v0.7.9.1) for insertions and deletions. The basic annotations of the called variants was done with annovar (v2013-08-23).<br>Full codes of all R scripts are available on request. All other statistics were done using GraphPad prism 7.0

For manuscripts utilizing custom algorithms or software that are central to the research but not yet described in published literature, software must be made available to editors/reviewers. We strongly encourage code deposition in a community repository (e.g. GitHub). See the Nature Research guidelines for submitting code & software for further information.

Data

Policy information about availability of data

All manuscripts must include a data availability statement. This statement should provide the following information, where applicable:

- Accession codes, unique identifiers, or web links for publicly available datasets
- A list of figures that have associated raw data
- A description of any restrictions on data availability

Gene expression data that support the findings of this study has been deposited in the Gene Expression Omnibus repository and will be made available prior to publication (GSE129877). All additional data sets generated or analysed during this study are included in this published article (and its supplementary information files).

Field-specific reporting

Please select the one below that is the best fit for your research. If you are not sure, read the appropriate sections before making your selection.

[x] Life sciences [ ] Behavioural & social sciences [ ] Ecological, evolutionary & environmental sciences

For a reference copy of the document with all sections, see nature.com/documents/nr-reporting-summary-flat.pdf

Life sciences study design

All studies must disclose on these points even when the disclosure is negative.

Sample size	Sample size was calculated with the help of a biostatistician using R version 3.4.0. Assumptions for power analysis were as follows: alpha error: 5%; beta error: 20%. Values for standard deviation and differences between experimental groups were based on previous experiments (whenever a similar data type was available). In all other cases a pilot group size was used.
Data exclusions	Two NR RNA samples were excluded from Nanostring analysis due to failed QC. For CIBER sort analysis of pre-PD-1 inhibitor GBM tissue of R and NR patients, 6 samples were excluded as these were obtained more than 6.5 months before the start of PD-1 therapy. In case animals had to be sacrificed prior to the pre-defined endpoint (due to weight loss or other termination criteria), they were excluded from any downstream analysis.
Replication	Key experiments (ICB response, macrophage phenotyping by flow cytometry, PD-L1 inhibition,) were all performed at least twice and data from one representative experiment are shown in this manuscript. All other experiments were performed once with biological replicates or technical replicates (as specified in figure legend).
Randomization	Mice were matched into the groups according to age, sex and tumor size (measured by MRI or calliper) at the time of treatment start.
Blinding	Intracranial tumor experiments were performed in a blinded manner (MRI, flow cytometric analyses).

Reporting for specific materials, systems and methods

We require information from authors about some types of materials, experimental systems and methods used in many studies. Here, indicate whether each material, system or method listed is relevant to your study. If you are not sure if a list item applies to your research, read the appropriate section before selecting a response.

Materials & experimental systems

n/a	Involved in the study	
[ ]	[x]	Antibodies
[ ]	[x]	Eukaryotic cell lines
[x]	[ ]	Palaeontology
[ ]	[x]	Animals and other organisms
[ ]	[x]	Human research participants
[x]	[ ]	Clinical data

Methods

n/a	Involved in the study	
[x]	[ ]	ChIP-seq
[ ]	[x]	Flow cytometry
[ ]	[x]	MRI-based neuroimaging

Antibodies

Antibodies used

Antigen conjugate, clone, distributor, catalogue number, LOT number

in vivo antibodies
mCD4 n.a., GK1.5, BioXCell, BE0003-1, LOT: 5730-2-7-8/0915
mCD8a n.a., 2.43, BioXCell, BE0061, LOT: 624616A2

mCSF1R n.a., AFS98, BioXCell, BE0213, LOT: 628217M1
mCTLA-4 n.a., 9D9, BioXCell, BE0164, LOT: 5632-4/1015 / 5987-316A1 / 636317D1 / 636317J1 / 636317J2
mPD-1 n.a., RMP1-14, BioXCell, BE0146, LOT: 614616D1 / 640517M2B / 5792X2/1015 / 640517M1B / 665418F1 / 695318A1
mPD-L1 n.a., 10F.9G2, BioXCell, BE0101, LOT: 665717O1

in vitro stimulation antibodies
hamster IgG n.a., n.a., MP Biomedicals, 0855397
mCD3e n.a., 145-2C11, eBioscience, 14-0031, LOT: 4310230
mCD28 n.a., 37.51, eBioscience, 14-0281, LOT: 4279532

immunohistochemistry antibodies
m/hCD3e n.a., A0452, DAKO, A0452, LOT: 20049827

flow cytometry antibodies
mCD3 APC-Cy7, 17A2, BioLegend, 1100222, LOT: B207497
mCD3 BV711, 17A2, BioLegend, 1100241, LOT: B245637
mCD3 FITC, 17A2, BioLegend, 100204, LOT: B221808
mCD3 PE, 17A2, BioLegend, 100206, LOT: 237806
mCD4 APC, RM4-5, BioLegend, 100516, LOT: B218256
mCD4 PB, RM4-5, BioLegend, 1100222, LOT: B207497
mCD4 PE Texas Red, RM4-5, Invitrogen, MCD0417, LOT: 1837385
mCD8 AF700, 53-6.7, BioLegend, 100730, LOT: B246153
mCD8 PE-Cy7, 53-6.7, eBioscience, 25-0081, LOT: E07510-1635
mCD8 PerCP-Cy7, 53-6.7, eBioscience, 45-0081, LOT: 4291993
mCD16/32 n.a. (block), 93, eBioscience, 14-0161 LOT: 4333612
mCD11b APC, M1/70, BioLegend, 101212, LOT: B226978
mCD11b BV421, M1/70, BioLegend, 101251
mCD11b FITC, M1/70, BioLegend, 101206, LOT: B224362
mCD11c BV421, N418, BioLegend, 117330, LOT: B209020
mCD25 PE, PC61, BioLegend, 102008, LOT: B19152
mCD25 PE-Cy7, PC61, BioLegend, 102016, LOT: B229046
mCD45 BV510, 30-F11, BioLegend, 103137, LOT: B260403 / B240739
mCD80 PE-Cy7, 16-10A1, BioLegend, 104734, LOT: B230008
mCD80 PerCP-eFluor710, 16-10A1, eBioscience, 46-0801, LOT: 1928918
mCCR2 APC, 475301, R&D, FAB5538A-025, LOT: VABLE0314121
mCCR4 PE, 2G12, BioLegend, 131203, LOT: B188734
mCCR5 PerCP-eFluor710, HM-CCR5, eBioscience, 46-1951, LOT: 4276802
mCCR6 PE-Cy7, 29-2L17, BioLegend, 129815, LOT: B252582
mFOXP3 APC, FJK-16s, eBioscience, 17-5773, LOT: 4330883
mFOXP3 FITC, FJK-16s, Invitrogen, 115773-8, LOT: 430671
mI-A/I-E PE-Cy7, M5/114.15.2, eBioscience, 25-5321, LOT: E14766-105
mPD-L1 PE, 10F.9G2, BioLegend, 124308, LOT: B246734
mPD-L1 PE-Cy7, MIH5, eBioscience, 25-5982, LOT: 4293382
fixable viability dye eFluor780, n.a., eBioscience, 65-0865, LOT: 1977883
fixable viability dye zombie red, n.a., BioLegend, 423109, LOT: B225035
mCD4 BV650, RM4-5, BioLegend, 100546, LOT: B223977
mCD8a BUV805, 53-6.7, BD Bioscience, 564920, LOT: 8157966
mCD11b BV711, M1/70, BioLegend, 101242, LOT: B235643
mCD11b BUV737, M1/70, BD Bioscience, 564443, LOT: 8206860
mCD11c PE-Cy5.5, N418, eBioscience, 35-0114-82, LOT: 1955296
mCD38 PE-Dazzle594, 90, BioLegend, 102729, LOT: B244188
mCD44 BV570, IM7, BioLegend, 103037, LOT: B252689
mCD45 BUV395, 30-F11, BD Bioscience, 564279, LOT: 8072723
mCD64 BV421, X54-5/7.1, BioLegend, 139309, LOT: B267417
mCD73 APC, TY/11.8, BioLegend, 127209, LOT: B222940
mCD80 BUV737, 16-10A1, BD Bioscience, 564670, LOT: 8240992
mCD86 Biotin, GL-1, BioLegend, 105004, LOT: B128959
mCD155 PE, TX56, BioLegend, 131507, LOT: B151035
mCD206 AlexaFlour700, C068C2, BioLegend, 141734, LOT: B253654
mF4/80 BV510, BM8, BioLegend, 123135, LOT: B271685
mIL-1b proform APC, NJTEN3, eBioscience, 17-7114-80, LOT: 4291569
mLy-6C FITC, AL-21, BD Bioscience, 553104, LOT: 5281566
mLy-6G BUV563, 1A8, BD Bioscience, 565707, LOT: 8318712
mMERTK PE-Cy7, DS5MMER, eBioscience, 25-5751-82, LOT: 1988685
mPD-1 BV785, 29F.1A12, BioLegend, 135225, LOT: B251641

	mPD-L1 BV605, 10F.9G2, BioLegend, 124321, LOT: B245034<br>mPD-L1 PE, MIH5, eBioscience, 12-5982-81, LOT: 4276911<br>mPD-L2 biotin, TY25, BioLegend, 107203, LOT: B230798<br>mTCRb PE-Cy5, H57-597, BioLegend, 109209, LOT: B271048<br>mTNFa BV421, MP6-XT22, eBioscience, 506328, LOT: B252150<br>mI-A/I-E BB700, M5/114.15.2, BD Bioscience, 746197, LOT: B189842<br>mCD16/32 BV711, 93, BioLegend, 101337, LOT: B251800<br>mNK1.1 BV785, PK136, BioLegend, 107203, LOT: B250369<br>mIL10 BV605, JES5-16E3, BioLegend, 505031, LOT: B233971<br>streptavidin BUV496, n.a., BD Bioscience, 564666, LOT: 564666
Validation	Magnetic sorting and in vitro stimulation antibodies have been titrated and established in previous experiments. Validation data of flow cytometry and immunohistochemistry antibodies can be found on the suppliers' website, e.g. in technical data sheets. Antibodies against critical and rare antigens have been titrated previously using positive control cells. In vivo antibodies have been validated repeatedly in previous experiments.

Eukaryotic cell lines

Policy information about cell lines

Cell line source(s)	Murine glioma cell line GL261 was purchased from National Cancer Institute (NCI<br>Tumor Repository). Murine melanoma cell line B16 was provided by G. Hämmerling (Molecular Immunology, German Cancer Research Center, Heidelberg, Germany).
Authentication	highthroughput Multiplex human Cell Authentification test (MCA), Castro et al.<br>2012 was not applicable for Gl261 cell line
Mycoplasma contamination	Cell lines were tesed negative for mycoplasma contamination regularly and before in vivo use. Highthroughput Multiplex Cell contamination (McCT), Schmitt M. et al. 2009
Commonly misidentified lines<br>(See ICLAC register)	no commonly misidentified lines from the ICLAC register were used in this study

Animals and other organisms

Policy information about studies involving animals; ARRIVE guidelines recommended for reporting animal research

Laboratory animals	C57BL/6J wild-type (WT) mice were purchased from Charles River or Janvier Labs. Sex- and age-matched mice were used for further experiments. If not stated otherwise, female mice were used for the experiments. All mice were 7-12 weeks of age at use. Mice were kept under SPF conditions at the animal facility of the DKFZ Heidelberg.
Wild animals	the study did not involve wild animals
Field-collected samples	the study did not involve samples collected from the field
Ethics oversight	Animal experiments were performed according to the rules of the German Animal Welfare Act and were licensed by the regional authority Karlsruhe. This study did not involve any human material.

Note that full information on the approval of the study protocol must also be provided in the manuscript.

Human research participants

Policy information about studies involving human research participants

Population characteristics	RNA sequencing data of a previously published patient cohort was analyzed (Zhao J et al., Nat. Med., 2019)
Recruitment	RNA sequencing data of a previously published patient cohort was analyzed (Zhao J et al., Nat. Med., 2019)
Ethics oversight	RNA sequencing data of a previously published patient cohort was analyzed (Zhao J et al., Nat. Med., 2019)

Note that full information on the approval of the study protocol must also be provided in the manuscript.

Flow Cytometry

Plots

Confirm that:

- [x] The axis labels state the marker and fluorochrome used (e.g. CD4-FITC).
- [x] The axis scales are clearly visible. Include numbers along axes only for bottom left plot of group (a 'group' is an analysis of identical markers).
- [x] All plots are contour plots with outliers or pseudocolor plots.
- [x] A numerical value for number of cells or percentage (with statistics) is provided.

Methodology

Sample preparation	Murine B16 tumors or Gl261-containing brain hemispheres were excised, washed in HBSS (Sigma-Aldrich) and cut into small pieces before tissue disruption in HBSS supplemented with 50 µg/ml Liberase D for 0,5 h under slow rotation at 37 °C. Dispersed tissue was mashed through a 100µm and 70 µm cell strainer and lymphocytes. For Gl261 sampleas, myelin removal was performed using myelin removal beads II (Miltenyi Biotec; 130-096) or by percoll density gradient as described in online methods. Murine splenocytes were isolated by homogenization using a cell strainer and ACK lysis. CD3+ and CD11b+ cells were purified with the MagniSort™ Mouse T cell Enrichment Kit (eBioscience; 8802-6820), MagniSort™ Mouse CD3 Positive Selection Kit (eBioscience; 8802-6840) or by MagniSort™ Mouse CD11b Positive Selection Kit (eBioscience; 8802-6860-74) on isolated cells using MACS according to manufacturer's instructions. In some cases, as described in the online methods, cells were treated with Brefeldin A to prevent secretion of cytokines, chemokines, and other secretory proteins before analysis. If required, single cell suspension were labeled with CellTrace Far Red according to the manufacturer's instructions (see online methods).
Instrument	FACS Canto II (BD Biosciences, V96300305), Attune NxT (Thermo Fisher Scientific, 2AAS232591116) or FACS Symphony (BD Biosciences)
Software	Data was collected with FACS Diva Software (version 9, BD Biosciences) or Attune NxT Software version 2.5. Data was analysed with Flow Jo Version 9 or 10 or FlowSOM.
Cell population abundance	CD8+ T cells and CD45highCD11b+ myeloid cells from murine Gl261 tumor samples were >95% CD8+ or >95% CD45highCD11b+ post sort, respectively
Gating strategy	Lymphocytes were defined by size and granularity in FSC-A vs. SSC-A plots. Subsequently, duplets were excluded in FSC-A vs FSC-H plots and dead cells were excluded by means of fixable viability dye positivity (except for apoptosis analysis). T cells were gated according to the lineage marker CD45 and CD3, then T cell types were gated in CD4 vs CD8 plots. Infiltrating Gl261 CD11b myeloid cells were gated as CD45highCD11b+, microglia as CD45lowCD11b+ of living single cells in CNS samples. Gating strategies of multi-parameter Gl261 TIL analysis is shown in Extended Data Figure 3. Boundaries between positive and negative cells were defined by use of fluorescence minus one (FMO) controls. Expression of cytokines, chemokine receptors and pro- and anti-inflammatory mediators was determined by calculating the median or mean fluorescence intensity.

- [x] Tick this box to confirm that a figure exemplifying the gating strategy is provided in the Supplementary Information.

Magnetic resonance imaging

Experimental design

Design type	Block design. All animals within one experiment were subjected to MRI on the same day.
Design specifications	Animals were subjected to MRI 3 times during one experiment at inervals between 6 and 7 days. I.e. MRI was performed on days 13, 19, and 26 post surgery if not otherwise stated in the online methods and figure legends.
Behavioral performance measures	n/a.

Acquisition

Imaging type(s)	structural T2 and T1-weighted structural magnetic resonance imaging
Field strength	9.4 Tesla small animal MRI
Sequence & imaging parameters	T2w: Turbo spin echo, coronal aquisition , matrix size 256x256, TE 33ms, TR 2500ms, two averages, flip angle 90°, resolution; 78µm x 78µm, slice thickness 0.7mm.<br><br>T1w (after iv administration of 0.01mmol Gadoteric acid : RARE, coronal aquisition, matrix size 200x200, TE 6ms, TR 1000ms, two averages, flip angle 90°, refocusing angle 180°, resolution; 100µm x 100µm, slice thickness 0.7mm.
Area of acquisition	whole brain scan

Diffusion MRI [ ] Used [x] Not used

Preprocessing

Preprocessing software	image files were exported as dicom files and segmentation of tumor volumes was performed in Osirix or Amira imaging software (version 4.12; Pixmeo) by manual segmentation.
Normalization	For assessing the contrast media uptake in the tumor, normalization was performed to the non-affected, contralateral side. For structural measurements, normalization was not performed.
Normalization template	no normalization template was used.
Noise and artifact removal	artifact or noise removal was not performed
Volume censoring	Osirix imaging software (version 4.12; Pixmeo) or Amira (FEI). Volumes were exported to microsoft excel. No censoring was performed

Statistical modeling & inference

Model type and settings	Machine-learning - Gradient boosting in R version 3.5.1 [R Foundation for Statistical Computing, Vienna, Austria] using the caret library. Tuning parameters [boosting iterations, max tree depth, shrinkage and min. terminal node size] were automatically optimized via resampling procedures.
Effect(s) tested	Predictive modeling of treatment failure (i.e. prediction of progression yes vs. no) at MRI3 from imaging data of previous timepoints.

Specify type of analysis: ☐ Whole brain ☒ ROI-based ☐ Both

Anatomical location(s)	Mice data was segmented on T2-weighted MRI data using Osirix (version 4.12; Pixmeo) or Amira (FEI). The ROI was delineated manually for every slice, and included the whole intracranial/intraparenchymal portion of the tumor in the mouse brain.
Statistic type for inference (See Eklund et al. 2016)	Radiomic data was analysed up to a voxel basis.
Correction	n/a

Models & analysis

n/a	Involved in the study
☒	☐ Functional and/or effective connectivity
☒	☐ Graph analysis
☐	☒ Multivariate modeling or predictive analysis

Multivariate modeling and predictive analysis

Radiomic features were calculated from the tumor segmentation masks using the medical imaging interaction toolkit (MITK, www.mitk.org). This included 146 first-order features, 33 volume and shape features, 200 texture features and 44 curvature features.

After the extraction of Radiomic data from the segmented MRI, predictive modeling from Radiomic data was performed using a gradient boosting machine-learning algorithm (implemented using R version 3.5.1 [R Foundation for Statistical Computing, Vienna, Austria] and the caret library) which iteratively constructs an ensemble of weak decision tree learners through boosting to form a single strong predictive model (the tuning parameters [boosting iterations, max tree depth, shrinkage and min. terminal node size] were automatically optimized via resampling procedures).

The performance of the gradient boosting classifier was assessed based on a two-times repeated 5-fold cross validation resampling procedure. The held-out predictions in each of the resampling iterations were used to calculate the accuracy, area under the ROC, sensitivity, specificity, no-information rate (largest class percentage for each molecular parameter i.e. the prediction or accuracy by chance) and a hypothesis test (using the binom.test function in R) to evaluate whether the accuracy rate is greater than the no-information rate. P values <0.05 were considered significant.
